# Hypothalamic FTO upregulates BDNF to promote GnRH expression through the PI3K/Akt pathway, leading to precocious puberty

**DOI:** 10.3389/fendo.2025.1665391

**Published:** 2025-10-31

**Authors:** Shaolian Zang, Yang Ouyang, Pin Li, Xiaoqin Yin

**Affiliations:** ^1^ Department of Endocrinology, Shanghai Children’s Hospital, School of Medicine, Shanghai Jiao Tong University, Shanghai, China; ^2^ Department of Urology, Shanghai Children’s Hospital, School of Medicine, Shanghai Jiao Tong University, Shanghai, China

**Keywords:** fat mass and obesity-associated (FTO), puberty onset, gonadotropin-releasing hormone (GnRH), brain-derived neurotrophic factor (BDNF), m6A methylation

## Abstract

**Background:**

Puberty is among the most important stages in human development. Timely activation of hypothalamic gonadotropin-releasing hormone (GnRH) neurons underlies the initiation of pubertal development. The fat mass and obesity-associated (FTO), which is expressed in the hypothalamus, may regulate the m6A methylation of its target genes, influencing GnRH expression in the hypothalamus during puberty onset. This study aimed to explore the mechanism by which FTO regulates the function of its target neurotrophins within the hypothalamus and to clarify the molecular pathway underlying precocious puberty.

**Methods:**

Methylated RNA immunoprecipitation sequencing (MeRIP-seq) was used to assess m6A methylation in the hypothalamus of female rats. Serum brain-derived neurotrophic factor (BDNF) levels were measured in girls with central precocious puberty (CPP) and matched controls. BDNF was applied to GnRH neurons *in vitro*. The functions of FTO were assessed through overexpression and knockdown in animal models, with downstream signaling evaluated via BDNF/PI3K/Akt pathway analysis. Intracerebroventricular (ICV) injections of a BDNF-overexpressing lentiviral vector or a negative control (NC) were used to investigate the role of central BDNF in regulating puberty onset and reproductive function.

**Results:**

Hypothalamic GnRH and FTO expression progressively increased whereas m6A methylation decreased during puberty. MeRIP-seq revealed significantly reduced m6A methylation of Bdnf mRNA in the arcuate nucleus (ARC) of female rats during early puberty. In the ARC, BDNF was expressed adjacent to GnRH-positive fibers and terminals. In addition, serum BDNF levels were higher in girls with CPP girls than in the control group. *In vitro*, BDNF treatment stimulated GnRH expression in GT1–7 cells. FTO positively regulated BDNF expression in an m6A methylation-dependent manner. FTO overexpression activated BDNF/PI3K/Akt signaling in the ARC and accelerated puberty onset. Conversely, FTO knockdown delayed vaginal opening and suppressed BDNF/PI3K/Akt signaling. ICV delivery of a BDNF-overexpressing lentivirus increased hypothalamic BDNF and GnRH expression, leading to a distinct endocrine profile.

**Conclusion:**

These findings suggest that FTO regulates the m6A demethylation of BDNF and promotes GnRH expression through the BDNF/PI3K/Akt signaling pathway. This study improves our understanding of the function of BDNF, which is critical for the development of therapeutic strategies for preventing and treating of precocious puberty.

## Introduction

Adolescence is an important period of transition from childhood to adulthood. The onset of puberty is a result of the timely activation of gonadotropin-releasing hormone (GnRH) neurons. Central precocious puberty (CPP) is a common disease characterized by abnormal sexual development. The primary cause of CPP is the premature stimulation of GnRH neurons and the hypothalamic–pituitary–gonadal axis (HPGA), which eventually leads to advanced pubertal development and sexual maturation (1). The same programmed process occurs during HPGA initiation and sexual maturation as during normal pubertal development. Recently, the prevalence of CPP has been increasing. Moreover, the frequency of this disease in females exceeds that in males and has a serious effect on the physiology and psychology of girls (2). Studies have shown that children with CPP are more likely to develop malignancies such as breast and gastrointestinal cancer, along with developing diabetic tendencies and obesity during adulthood (3). Therefore, elucidating the mechanisms that regulate the release of GnRH is essential for understanding the initiation of puberty. The onset of puberty is directed by a complex interplay among neurons and glial cells located within the hypothalamus. Neurons and glial cells drive puberty onset and sexual development by promoting a pulsing increase in GnRH secretion, leading to sexual maturity. The increase in pulsing GnRH secretion is attributed to the gradual elimination of prepubertal inhibition and increased responsiveness to excitatory effects. The inhibitory factor mainly includes dynorphin. Excitatory factors mainly include Kiss1, neurokinin B (NKB), and leptin (4). Previous studies have shown a functional connection between neurotrophic factors including BDNF in the ovaries and the Kiss1 receptor signaling system (5).

Neurotrophins (NTs) are secreted peptides or proteins produced by the body that act on the nervous system. They influence the development, differentiation and survival of both neurons and glial cells (6). NTs include nerve growth factor (NGF), brain-derived neurotrophic factor (BDNF), neurotrophin-3 (NT-3), neurotrophin-4 (NT-4), and other neuropeptides of nonmammalian origin. The various functions of NTs are carried out mainly through their binding to specific receptors (7). Brain-derived neurotrophic factor (BDNF) is a 27 kDa protein belonging to the neurotrophic factor superfamily and is expressed in various tissues and organs throughout the body. Its structure is complex, comprising several regulatory elements and four distinct promoters (8). A previous study of primary cultures of GnRH cells from mouse embryos revealed that BDNF can induce GnRH neurite growth (9). Cumulus cells can also secrete BDNF, which promotes the maturation of mouse oocytes (10, 11). This effect is mediated by high-affinity neurotrophin receptor kinase 2 (NTRK2) expressed in oocytes. When ligands trigger receptor activation, the maturation of fertilized eggs is facilitated via activation of phosphatidylinositol 3-kinase (PI3K) signaling (12). BDNF is the most abundant, widely distributed, and complex neurotrophic factor in the mammalian brain and serves as a crucial protein. The presence of BDNF-positive neurons in the hypothalamic arcuate nucleus (ARC), ventromedial nucleus (VMN), dorsomedial nucleus (DMN), paraventricular nucleus (PVN), and median eminence (ME) has been confirmed (13, 14). These regions are also responsible for integrating and encoding information related to energy homeostasis in the body and are closely related to HPGA initiation (15). Furthermore, administering the recombinant BDNF protein directly to the central nervous system can influence the activity of HPGA in sheep, primarily by inducing the expression of the GnRH mRNA (16). Consequently, a plausible hypothesis is that BDNF plays a role within the core neuroendocrine circuit to regulate HPGA function.

Interestingly, hippocampal BDNF expression decreases in mice deficient in the fat mass and obesity-associated (FTO) protein, suggesting that FTO has a regulatory effect on the BDNF pathway (17). As the key ‘eraser’ of N6-methyladenosine (m6A) methylation, FTO has been shown to modulate energy metabolism in the hypothalamic ARC (18–20). However, the roles of BDNF signaling and FTO-mediated m6A demethylation of BDNF in pubertal development remain inadequately defined. In our research, we further investigated the mechanism by which FTO-driven m6A demethylation functions as a regulatory system at the onset of puberty. FTO levels increased, while m6A methylation gradually decreased with the progression of puberty. In pubescent female rats, m6A modification of Bdnf mRNA within the ARC significantly decreased, as evidenced by m6A epigenetic transcriptomic sequencing. We demonstrated that the FTO-catalyzed m6A methylation of BDNF modulates the expression of GnRH through the PI3K/Akt pathway in the hypothalamus, influencing puberty onset. Our data suggest a previously unrecognized link between FTO and BDNF signaling during puberty onset, which helps us to more fully understand pubertal development.

## Materials and methods

### Animals

Three-week-old and five-week-old female Sprague–Dawley rats were purchased from Shanghai Jie Sijie Experimental Animal Co., Ltd. (Shanghai, China). FTO+/- mice were purchased from Cyagen Biosciences (Suzhou, China). In this study, we used female mice aged 3 to 5 weeks for all the experiments. Each of the juvenile and pubertal groups included 13 female rats, among which 5 were used for real-time PCR, 5 for m6A detection, and 3 for immunofluorescence (IF) staining. A total of 9 female rats were included in the group with FTO overexpression within the ARC and the corresponding control group, among which 3 were used for real-time PCR, 3 for Western blot analyses, 3 for IF staining, and 6 out of 9 for histological assessments. The number of FTO wild-type and heterozygous mice were the same as those of the above-mentioned female rats. The animals were housed in an environment free from pathogens and lived in a cage with a controlled temperature that cycled through 12 hours of light and 12 hours of darkness. This animal experiment was approved by the Animal Experiment Committee of Shanghai Children’s Hospital (SHCH-IACUC-2022-XMSB-40). Institutional guidelines and the guidelines of the Committee for the Purpose of Control and Supervision of Experiments on Animals (CPCSEA) were strictly followed for animal handling, and the reporting of animal experiments follows the ARRIVE (Animal Research: Reporting of *In vivo* Experiments) guidelines.

### Participants

From July to September 2022, peripheral serum samples were obtained from 14 patients with CPP and 10 healthy girls for the determination of BDNF levels. The inclusion and exclusion criteria included the onset of breast development before the age of 8 years in girls and a greater-than-normal annual growth rate. Patients with peripheral precocious puberty (PPP) were not included. This study was approved by the Medical Ethics Committee of Shanghai Children’s Hospital, School of Medicine, Shanghai Jiao Tong University, and informed consent was obtained from all participants (approval number: 2020R153-E02).

### ELISA

Plasma levels of brain-derived neurotrophic factor (BDNF) in patients were measured quantitatively using an enzyme-linked immunosorbent assay (ELISA) kit according to the manufacturer’s instructions (Multi Sciences, China). The range of BDNF concentrations was 6.25 pg/mL–400 pg/mL. Whole blood samples were collected from the hearts of female rats, and plasma was obtained after centrifugation. The levels of E2, LH, and FSH in female rat serum were measured with ELISA kits (USCN Life Science, USA) according to the manufacturer’s instructions. The experiments were conducted in triplicate.

### Tissue collection

Female rats and mice were administered sodium pentobarbital at concentrations of 3% or 1% (0.5 mL/100 g body weight) to induce anesthesia, respectively. The rats and mice were humanely sacrificed by cervical dislocation. Afterward, they were decapitated to obtain the entire brain. Based on the anatomical methods reported in the literature (21), we further collected arcuate nucleus (ARC) and hypothalamic tissues for subsequent molecular biological experiments. Total RNA was extracted for sequencing from the ARC, which were dissected from fresh mouse brain tissues. The brain tissues were rapidly frozen in liquid nitrogen and sectioned using a cryostat (Epredia HM525 NX, Shanghai, China). The ARC regions were carefully selected under a stereomicroscope based on anatomical landmarks. The ARC region was identified according to the third ventricle as the central reference point, delineating a triangular area extending bilaterally from the ventricle toward the arcuate nucleus. The anatomical location of the hypothalamus was mainly defined by four landmark structures, including the temporal sulci on either side, the posterior margin of the body of the papilla at the posterior boundary, and the anterior chiasma at the anterior boundary. The dissected fragments were approximately 2 mm thick. For qPCR, WB and m6A detection, the whole hypothalamus was isolated and frozen in liquid nitrogen.

### Quantitative real-time PCR

Total RNA was extracted from tissues and cells with TRIzol Reagent (Invitrogen, CA, USA). cDNA was synthesized from the extracted RNA (1 μg) via reverse transcription using a reverse transcription kit (AG, Hunan, China). Then, qRT–PCR was conducted with a qPCR Kit (AG, Hunan, China). β-Actin was validated as a stable internal control across developmental stages via consistency checks and comparisons to Gapdh expression. β-Actin was used as the internal control, and relative gene expression was determined using the 2-^△△Ct^ method. The sequences are listed in the **Supplementary Materials** (**Supplementary Table S1**).

### Western blotting

Protein was extracted with lysis buffer (Thermo, MI, USA) supplemented with protease and phosphatase inhibitors (Roche, CH). After quantification using a BCA Kit (Thermo, MI, USA), 30 μg of protein from each sample was separated using SDS–PAGE and transferred to membranes. The membranes were subsequently incubated with primary antibodies overnight at 4 °C. Afterward, the membranes were treated with an HRP-conjugated secondary antibody and incubated at room temperature for 1 h. Visualization was achieved with an enhanced chemiluminescence (ECL) system. The protein band intensities were analyzed using ImageJ software. The antibodies used are listed in the **Supplementary Materials** (**Supplementary Table S2**).

### Immunofluorescence staining

We conducted IF staining of the hypothalamus to compare the expression levels of BDNF, GnRH and FTO. The hypothalamus was cut into 20 μm sections and then placed in PBS. The tissues were incubated with primary antibodies for 24 h at 4 °C. Afterward, corresponding fluorescent dye-conjugated secondary antibodies were applied to the tissues and incubated for 1 h in the dark. We subsequently added 4’, 6-diamidino-2-phenylindole (DAPI) and an anti-fluorescence quencher to the tissues. Finally, images of cells with positive staining were captured using a fluorescence microscope and confocal microscope to analyze of the expression of the proteins of interest and distribution of positive cells. The specific parameter settings were as follows: the optical section thickness was 6 μm, and the total thickness (including tissue, slide and cover glass) was 0.9 - 1.2 mm. The excitation wavelength for green fluorescence was 488 nm, and that for red fluorescence was 555 nm.

### Methylated RNA immunoprecipitation sequencing

The ARC tissues of three rats were pooled into one sample to create one sequencing library for each group (n = 2 libraries in total: the prepubertal pooled group and the early pubertal pooled group). MeRIP-seq was performed by Cloudseq Biotech, Inc. (Shanghai, China). m6A RNA immunoprecipitation (IP) was performed with a GenSeq™ m6A-MeRIP Kit (GenSeq, Inc., Shanghai, China). RNA sequencing libraries were generated for both input samples for IP and m6A IP. The generated libraries were analyzed with an Illumina NovaSeq 6000 system using 150 bp paired-end reads. The reads were then analyzed with Cutadapt software (v1.9.3) after the removal of low-quality reads and 3’ adaptor trimming. GO analysis and KEGG pathway enrichment analysis of the genes encoded by the differentially methylated proteins was performed. Pathway analysis was performed using the GO and KEGG databases (22).

### Cell culture and treatment

GT1–7 cells were obtained from the Shanghai Clinical Center for Endocrine and Metabolic Diseases, Shanghai Jiao Tong University. All the cells used in the experiments were passaged between 15 and 25 times. The cells were cultured in Dulbecco’s modified Eagle’s medium (DMEM; Gibco, NY, USA) supplemented with 10% fetal bovine serum (FBS; Gibco), 100 U/mL penicillin and 100 μg/mL streptomycin. The cells were maintained at 37 °C in 5% CO_2_ in an incubator. Recombinant human BDNF (PeproTech, USA) was reconstituted in sterile PBS supplemented with 0.1% bovine serum albumin (BSA). When the cell confluence reached approximately 70–80%, the cells were treated with 100 ng/mL BDNF. After 24 hours, the cells were collected for the analysis of GnRH mRNA expression.

### RNA interference and plasmid transfection

OBiO Technology (Shanghai, China) designed and synthesized lentiviral constructs expressing BDNF, FTO, and mutant plasmids. Mutant FTO (FTO-MUT) was generated by introducing a point mutation (R96Q) at its enzymatic active site (23, 24). This mutation abolished its m6A demethylase activity while maintaining its protein expression and stability. After the plasmids and lentiviral vectors were successfully constructed and verified by sequencing, they were transfected into the cells. Under a fluorescence microscope, the transfection conditions that led to a higher transfection rate were dentified by observing the expression of GFP fluorescence. Transfection was subsequently performed again under the identified optimal conditions, and the expression levels of the target genes and proteins were further verified by qPCR and Western blotting, respectively. Cells were transfected with lentiviruses and plasmids using Lipofectamine 3000 (Invitrogen, USA). After 48 h or 72 h of transfection, the cells were collected. An EFFS or CMV promoter was introduced into the scAAV expression vector to clone rat FTO cDNA. scAAV-FTO and scAAV-control were obtained from Vigene Biosciences (Shandong Vigene Biosciences Co., Ltd., China). The final titer was 5.24 × 10^^13^-8.01 × 10^^13^ vg/mL. The sequences are listed in the **Supplementary Materials** (**Supplementary Table S3**).

### m6A qPCR

Total RNA was extracted using an RNA Extraction Kit (AG, China), and DNase I was used to eliminate DNA contamination. mRNA was then purified from the RNA samples using a Dynabeads™ mRNA purification kit (Thermo Fisher, USA). The EpiQuik™ CUT&RUN m6A RNA Enrichment Kit (Epigentek, USA) was subsequently used for m6A IP. The relative expression levels of m6A-methylated target genes were determined by calculating the Cq value of the m6A IP sample divided by the Cq value of the input. Detailed information about the primer sequences utilized can be found in the **Supplementary Materials** (**Supplementary Table S1**).

### Dual-luciferase reporter assay

The pMIR-REPORT luciferase vector was modified to include either the original or altered (substitution of m6A with T) version of the downstream Bdnf exon. Cells in 24-well plates were cotransfected with these constructs alongside OE-FTO (KD-FTO or control vector). Firefly and Renilla luciferase activities were measured in each well 24 hours after transfection using a dual-luciferase reporter assay system (Vazyme, China).

The sequence of the Bdnf exon with wild-type m6A-methylated sites as follows:

GAGCAAAGCC**GAACT**TCTCACATGATGACTTC**AAACAAGACA**CATTACCTTCCAGCATCTGTTGGGGAGACGAGATTTTA**AGACA**CTGAGTCTCCA**GGACA**GCAAAGCCAC

The sequence of the Bdnf exon with mutant m6A-methylated sites was as follows:

GAGCAAAGCC**GATCT**TCTCACATGATGACTTC**AATCAAGTCA**CATTACCTTCCAGCATCTGTTGGGGAGACGAGATTTTA**AGTCA**CTGAGTCTCCA**GGTCA**GCAAAGCCAC

### Stereotaxic injections

The brains of female rats aged 3 weeks were injected with scAAVs or a lentivirus (LV) via a precision-guided technique. First, they were anesthetized using a 1% solution of sodium pentobarbital (0.5 mL per 100 g of body). Each rat received a microinjection of 1 microliter of scAAV overexpressing FTO into the bilateral ARC (0.4 mm lateral and 1.6 mm posterior to bregma, 9.4 mm from the dural surface). The virus was injected at a rate of 250 nanoliters per minute using a finely calibrated 10 μL Hamilton syringe. Each rat also received an intracerebroventricular (ICV) microinjection of 40 μL of an LV overexpressing BDNF (1.5 mm lateral and 0.8 mm posterior to bregma, 3.5 mm from the dural surface). The LV was injected at a rate of 4 microliters per minute using a finely calibrated 50 μL Hamilton syringe. All surgical interventions were performed over approximately 20 minutes. In the recovery phase, the rats were kept on a heated pad within a sterile enclosure until they were fully conscious and could be returned to their home cages.

### Hematoxylin–eosin staining

After the mice were euthanized the intact uterus and ovaries were quickly removed. The wet weight was measured, and the ratio of wet weight to body weight was calculated to determine the organ coefficient of the uterus/ovary. The tissues were immediately placed in an adequate amount of 4% paraformaldehyde in phosphate buffer solution and fixed at room temperature for 12–24 hours. Afterward, the samples were dehydrated, cleared, and embedding in paraffin. Continuous sections were cut at a thickness of 4 μm. Afterward, the sections were placed in a 60 °C oven for 2 hours. After dewaxing, the sections were rinsed with running water for 2 minutes and sequentially stained with hematoxylin and eosin. Finally, the sections were analyzed under an optical microscope. We analyzed the HE-stained images of the largest cross-section of the ovaries of each female mouse. A follicle was counted only when its oocyte nucleus was clearly visible in the slice. This approach ensured that each follicle was counted only once at its maximum cross-section. The CL is a large and complex structure. Only slices of the central connective tissue core were considered to contain the CL. Using these counting methods, double counting of follicles and CLs was avoided.

### Statistics

Statistical analyses were conducted using GraphPad Prism 8.0 (GraphPad, San Diego, CA, USA) and SPSS software (version 20, SPSS, Inc., Chicago, IL, USA), and the results are presented as the mean ± standard errors of the means (S.E.M.s). Initially, the data were checked for normal distribution and homogeneity of variance. Student’s t test was employed to compare two sets of data that met these criteria in SPSS. Multiple comparisons were performed using one-way analysis of variance (ANOVA) followed by Tukey’s *post hoc* test. A *p* value less than 0.05 was considered to indicate statistical significance.

## Results

### MeRIP-seq of the hypothalamic ARC of 3-week-old and 5-week-old female rats

**Figure 1:**
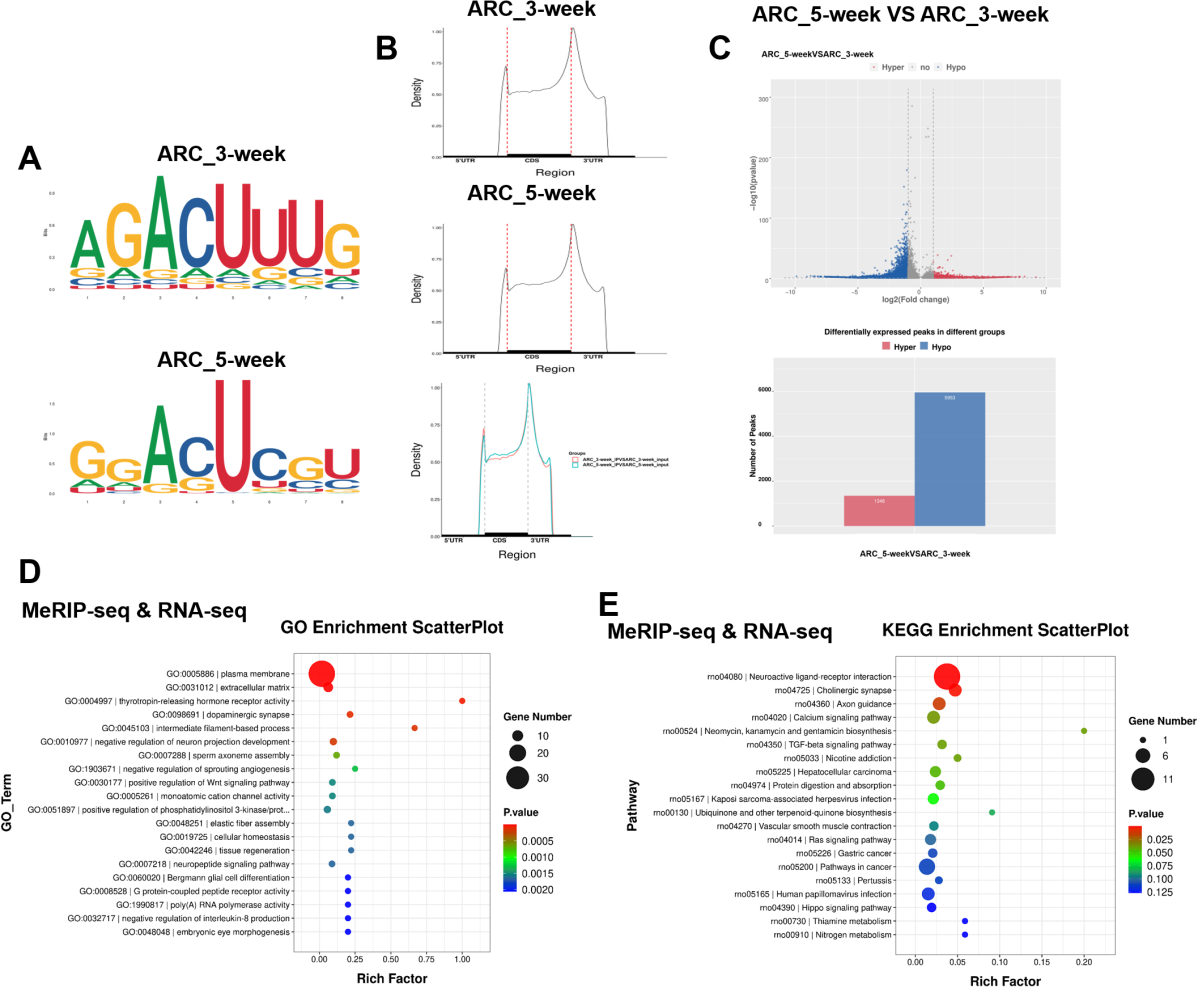
MeRIP-seq and RNA-seq of the hypothalamic ARC of 3-week-old and 5-week-old female rats. **(A)** Top enriched motifs for m6A peaks identified in the ARCs of female rats at 3 and 5 weeks of age. **(B)** Distribution of m6A sites plotted by mRNA transcript. **(C)** Volcano plot showing the m6A enrichment of genes in the ARCs of female rats at 3 and 5 weeks of age. **(D)** Gene Ontology (GO) enrichment analysis of differentially expressed genes (DEGs) in the ARCs of female rats at 3 and 5 weeks of age based on combined MeRIP-seq and RNA-seq data. **(E)** Kyoto Encyclopedia of Genes and Genomes (KEGG) pathway analysis of DEGs in the ARCs of female rats at 3 weeks and 5 weeks of age based on combined MeRIP-seq and RNA-seq data. ARC, arcuate nucleus (ARC_3-week, ARC_5-week; n = 1). Pathway analysis was performed using the KEGG database (www.kegg.jp/kegg/kegg1.html).

In female rats with normal development, the level of the m6A methylation decreased from the juvenile stage to early puberty (**Supplementary Figure S1A**). Both the mRNA and protein levels of FTO in the hypothalamus increased substantially upon puberty onset (**Supplementary Figures S1B, C**). Next, we performed MeRIP-seq of RNA extracted from the ARC of female rats. We analyzed differential expression based on the fold enrichment of the peaks in the two groups. m6A peaks were strongly enriched in the RRACH motif (**Figure 1A**) and were located primarily in coding regions (CDRs) and 3’ untranslated regions (UTRs), and near stop codons (**Figure 1B**; **Supplementary Figure S1D**). The distribution of genes with different abundance of m6A sites is displayed in **Supplementary Figure S1E**. Compared with those in the ARC of the hypothalamus in 3-week-old female rats, 1, 348 transcripts had increased m6A peak enrichment, and 5, 953 transcripts had fewer enriched m6A sites (**Figure 1C**). Gene Ontology (GO) enrichment analysis revealed that the differentially expressed genes were predominantly associated with a variety of signaling pathways, including the plasma membrane, extracellular matrix, thyrotropin-releasing hormone receptor activity, and phosphatidylinositol 3-kinase (PI3K)/protein kinase B signal transduction (**Figure 1D**). Kyoto Encyclopedia of Genes and Genomes (KEGG) pathway analysis indicated the substantial enrichment of the various differentially expressed genes in specific signaling pathways: neuroactive ligand–receptor interaction, cholinergic synapse, and calcium signaling (**Figure 1E**). Further GO (**Supplementary Figure S1F**) and KEGG (**Supplementary Figure S1G**) analyses of the DEGs identified using RNA-seq were also conducted. GO and KEGG enrichment analyses of the differentially expressed genes related to pubertal development revealed that compared with 3-week-old female rats, reduced m6A peaks and increased mRNA levels were observed for Bdnf in the ARC of 5-week-old female rats. Key genes associated with the PI3K/Akt pathway and neuroactive ligand–receptor interactions seemed to undergo m6A methylation, as indicated by GO and KEGG pathways enrichment analyses.

### BDNF expression is positively associated with GnRH expression during puberty

**Figure 2:**
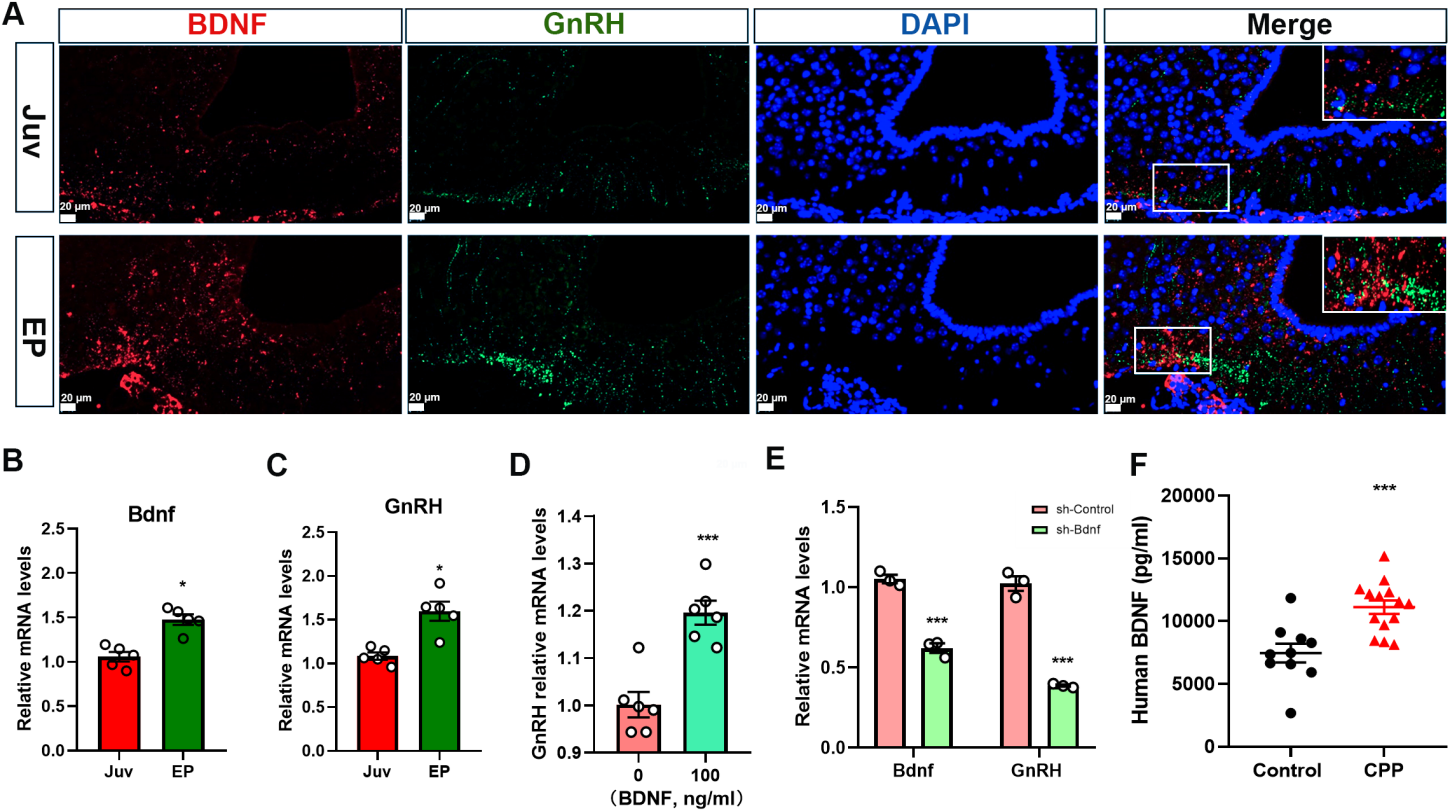
BDNF expression is positively correlated with GnRH expression during puberty. **(A)** Double immunofluorescence (IF) staining showed that the BDNF protein (red) was colocalized with the GnRH (green) protein and cell nuclei (DAPI, blue) in the arcuate nucleus (ARC) (n=3; scale bar: 20 μm, 50 μm). **(B)** qRT–PCR revealed that Bdnf mRNA expression in the hypothalamus increased gradually with time following puberty onset (n=5). Juv, juvenile, 3 weeks old; EP, early puberty, 5 weeks old. **(C)** qRT–PCR revealed that GnRH mRNA expression in the hypothalamus increased gradually with time following puberty onset (n=5). Juv, juvenile, 3 weeks old; EP, early puberty, 5 weeks old. **(D)** qRT–PCR revealed that GnRH expression was increased in GT1–7 cells stimulated with 100 ng/ml recombinant BDNF protein (n=6). **(E)** qRT–PCR showed that the GnRH mRNA level decreased when Bdnf expression was downregulated (n=3). **(F)** BDNF levels in the peripheral serum of girls with central precocious puberty (CPP) were higher than those in girls in the control group (control, n=10; CPP, n=14). The results are shown as the means ± S.E.M.s. *P < 0.05 and ***P < 0.001. Two-tailed Student’s t test was used.

In this experiment, using female rats at different normal developmental stages and samples form girls with CPP, we further investigated the role of BDNF in the initiation of puberty. Double IF staining was conducted to analyze the relationship between BDNF and GnRH across different developmental stages. Notably, BDNF expression was increased in the ARC of female rats during early puberty, and BDNF was expressed near GnRH-positive fibers (**Figure 2A**). GnRH cell bodies were located in the preoptic area (POA), which was anatomically distant from the ARC, this spatial arrangement suggested that the influence of BDNF originating from the ARC on GnRH fiber density and GnRH release was likely mediated indirectly. Concurrently, Bdnf mRNA expression also increased during early puberty (**Figure 2B**). A marked increase in GnRH mRNA levels in the hypothalamus was observed in tandem with the onset of puberty (**Figure 2C**). We further investigated the regulatory relationship between BDNF and GnRH by treating GT1–7 cells with 100 ng/ml recombinant BDNF protein. This treatment resulted in a significant increase in GnRH mRNA expression (**Figure 2D**). Additionally, we designed three RNA interference sequences targeting the mouse Bdnf gene and inserted them into a lentiviral vector to suppress BDNF expression (**Supplementary Figures S2A, B**). We selected shRNA-Bdnf-2 to effectively suppress Bdnf expression in subsequent experiments. Interestingly, BDNF knockdown reduced the GnRH mRNA level (**Figure 2E**). We recruited healthy controls and girls with CPP at our hospital to further investigate the serum level of BDNF in girls with CPP. The luteinizing hormone-releasing hormone (LHRH) test results indicated that the girls with CPP included in the present study exhibited early onset of HPGA activity. A comparison of the baseline data between the two groups is presented in **Supplementary Table S4**. Significant differences in body weight (*P* = 0.0314), BMI (*P* = 0.0382), and Tanner stage of breast development (*P* < 0.001) were observed between the two groups. We subsequently measured BDNF levels in peripheral serum using ELISA. BDNF levels were nearly two-fold higher in the peripheral serum of girls with CPP than in that of controls (**Figure 2F**). These data indicated that BDNF expression is positively associated with GnRH expression during puberty.

### FTO positively regulates BDNF expression by activating PI3K/Akt signaling

**Figure 3:**
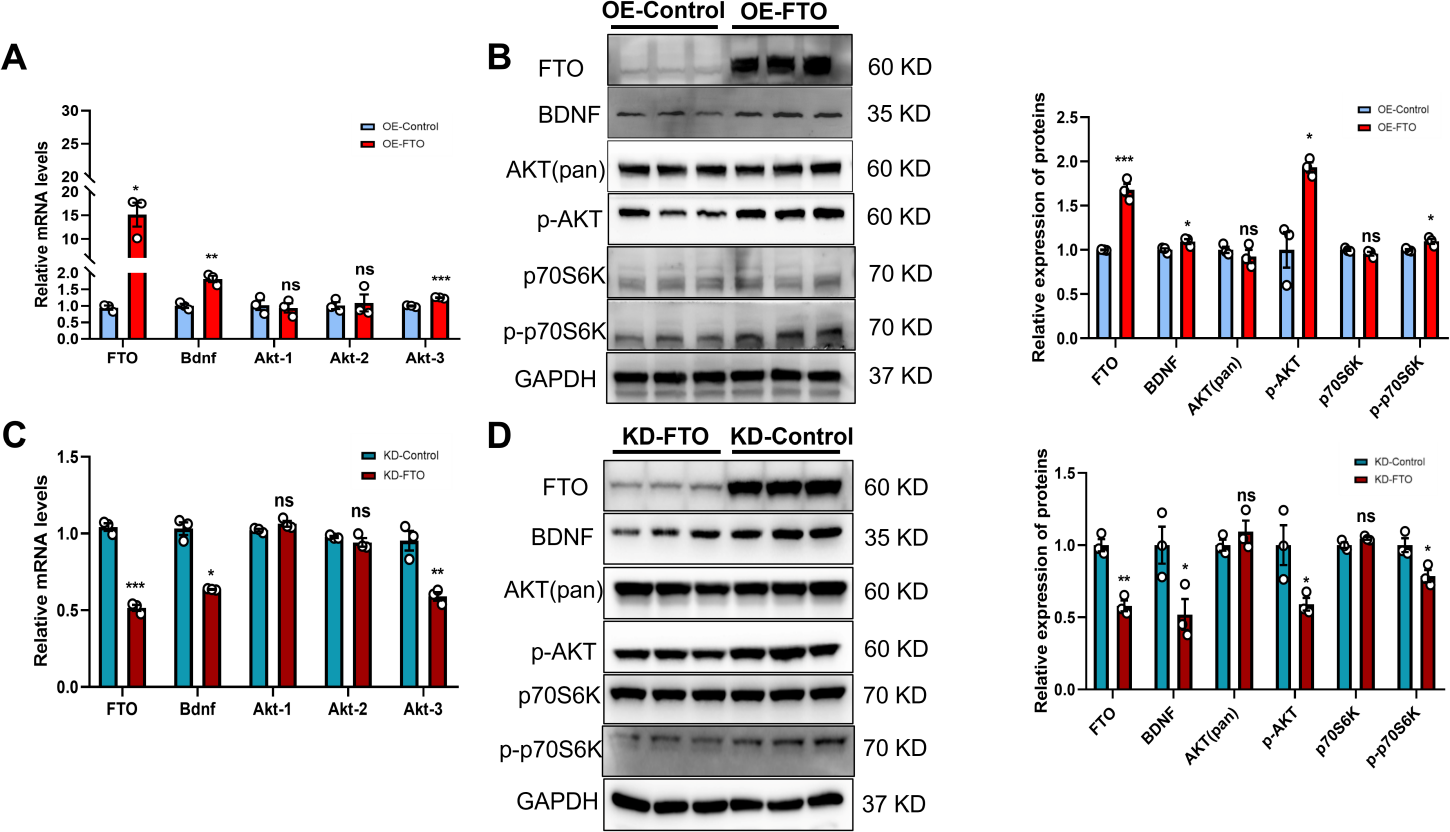
FTO positively regulates BDNF expression by activating PI3K/Akt signaling. **(A, C)** FTO, Bdnf, Akt-1, Akt-2 and Akt-3 mRNA levels in GT1–7 cells overexpressing FTO (OE-FTO) **(A)** or with knockdown of FTO (KD-FTO) **(C)** were determined via qRT–PCR. FTO positively regulated the expression of Bdnf (n=3). β-actin mRNA was used as an internal control. **(B)** Western blot showing that BDNF/PI3K/Akt signaling was activated following FTO overexpression (n=3). The protein levels of FTO, BDNF, p-AKT, and p-p70S6K were significantly increased, while total Akt and p70S6K levels did not change. **(D)** The expression of proteins in the BDNF/PI3K/Akt signaling pathway was reduced when FTO expression was downregulated (n=3). The protein levels of FTO, BDNF, p-AKT, and p-p70S6K were significantly decreased, while the total Akt and p70S6K levels did not change. GAPDH was used as a loading control. The bars represent the means ± S.E.M.s. *P < 0.05, **P < 0.01, ***P < 0.001, and ns, *P* > 0.05 compared with the control group. Two-tailed Student’s t test was used.

Based on the above results, we further explored the regulatory relationship between BDNF and the m6A demethylase FTO using GT1–7 cells. Transcripts encoding various components of the BDNF signaling pathway were found to undergo m6A methylation at 3 and 5 weeks. qRT–PCR revealed increased mRNA levels of Bdnf and Akt-3 in GT1–7 cells stably overexpressing FTO (OE-FTO) (**Figure 3A**). We subsequently conducted Western blot analysis. The results indicated a significant increase in BDNF levels in GT1–7 cells overexpressing FTO. The levels of phosphorylated Akt (p-Akt), a downstream target of BDNF, were increased in OE-FTO cells, whereas total Akt levels remained unchanged. Additionally, the total level of p70S6K, another component of the BDNF signaling pathway, did not change. However, compared with control cells, OE-FTO cells presented increased levels of phosphorylated p70S6K (p-p70S6K) (**Figure 3B**). The stable knockdown of FTO in GT1–7 cells resulted in a reduction in the mRNA levels of both Bdnf and Akt-3 (**Figure 3C**). Furthermore, when FTO expression decreased, BDNF levels decreased significantly. The levels of total Akt and p70S6K remained stable. However, the levels of p-Akt and p-p70S6K were significantly decreased (**Figure 3D**). Collectively, these findings suggest that variations in FTO expression may influence the functionality of BDNF signaling.

### FTO modulates BDNF expression in an m6A methylation-dependent manner

**Figure 4:**
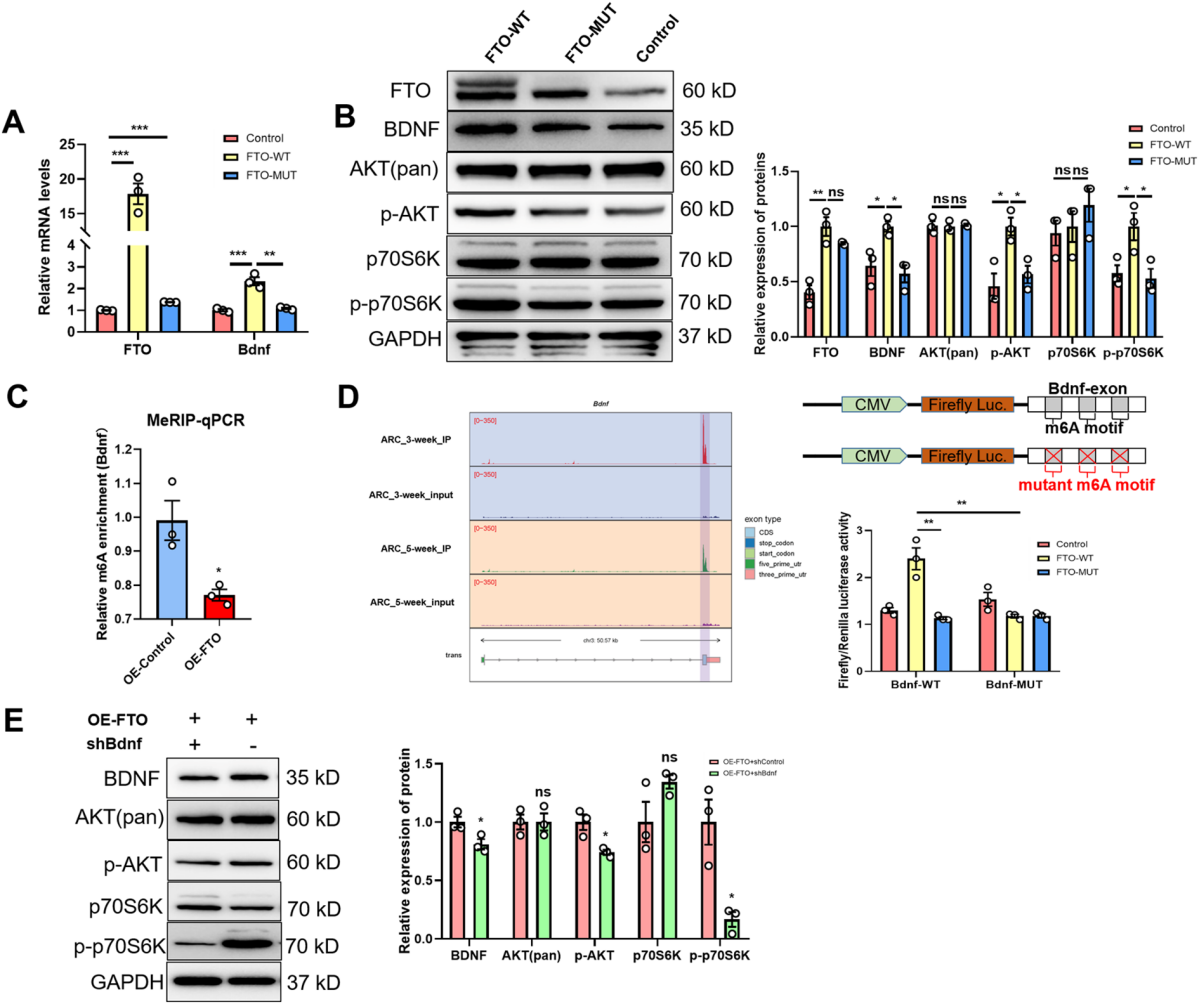
The m6A demethylase FTO regulates Bdnf expression in an m6A methylation-dependent manner. **(A)** qRT–PCR analysis showing that the m6A demethylase FTO regulates Bdnf expression in an m6A methylation-dependent manner (n=3). **(B)** Representative Western blot images showing the levels of the FTO protein and proteins in the BDNF/PI3K/Akt signaling pathway. The protein levels of FTO, BDNF, p-AKT, and p-p70S6K were significantly increased in FTO wild-type cells, while total Akt and p70S6K levels did not change. GAPDH was used as a loading control when WT- or MUT-FTO was ectopically expressed (n=3; one-way ANOVA). **(C)** Methylated RNA immunoprecipitation (MeRIP)-qPCR analysis of m6A-modified Bdnf mRNA revealed that the m6A methylation level was lower in FTO-overexpressing (OE) cells than in control cells (n=3; two-tailed Student’s t test). **(D)** Left panel: Gene tracks based on the MeRIP-seq analysis of Bdnf using IGV in the ARCs of 3-week-old and 5-week-old female rats. Right panel: Relative luciferase activity of the WT and MUT (A- to T-mutation) Bdnf-exon luciferase reporters in control, WT-FTO-overexpressing and MUT-FTO-overexpressing cells (n=3; one-way ANOVA). Firefly luciferase activity was measured and normalized to Renilla luciferase activity. **(E)** Expression of the BDNF, p-AKT, p-p70S6K, total Akt and p70S6K proteins after Bdnf was knocked down in OE-FTO cells, as determined by Western blotting (n=3; two-tailed Student’s t test). The results are shown as the means ± S.E.M.s. *P < 0.05, **P < 0.01, ***P < 0.001, and ns, *P* > 0.05.

In this study, using GT1–7 cells, we further investigated the mechanism by which FTO regulates BDNF expressin *in vitro*. Our subsequent exploration focused on determining whether FTO plays a role in modulating BDNF levels through RNA demethylation. Our findings confirmed this hypothesis, as the expression of Bdnf mRNA increased significantly upon the overexpression of FTO-WT but not FTO-MUT (**Figure 4A**). Moreover, FTO-WT, but not FTO-MUT or a control vector, significantly increased the levels of the BDNF, p-Akt and p-p70S6K proteins (**Figure 4B**). However, the levels of total Akt and p70S6K remained unchanged. Gene-specific MeRIP–qPCR assays revealed a significant reduction in the m6A methylation of Bdnf mRNA following FTO overexpression (**Figure 4C**). We performed mutagenesis and dual-luciferase reporter assays to elucidate the critical role of m6A methylation in the regulation of target mRNAs by FTO. Our m6A-seq data indicated the m6A methylation of the exon of Bdnf. Notably, Bdnf exhibited reduced m6A peaks in the ARC of 5-week-old female rats. The luciferase activity of reporter constructs containing wild-type exon fragments of Bdnf was significantly increased upon the overexpression of FTO-WT, whereas no increase was observed upon the overexpression of FTO-MUT. Furthermore, Bdnf expression was inhibited by an A- to T- mutation at m6A sites (**Figure 4D**). Additionally, we successfully transfected shRNA-Bdnf into GT1–7 cells overexpressing FTO. The results showed that the effects of FTO overexpression were significantly mitigated when BDNF expression was inhibited (**Figure 4E**). These results indicate that FTO modulates BDNF expression through an m6A methylation-dependent mechanism.

### FTO overexpression in the ARC activates BDNF signaling in female rats

**Figure 5:**
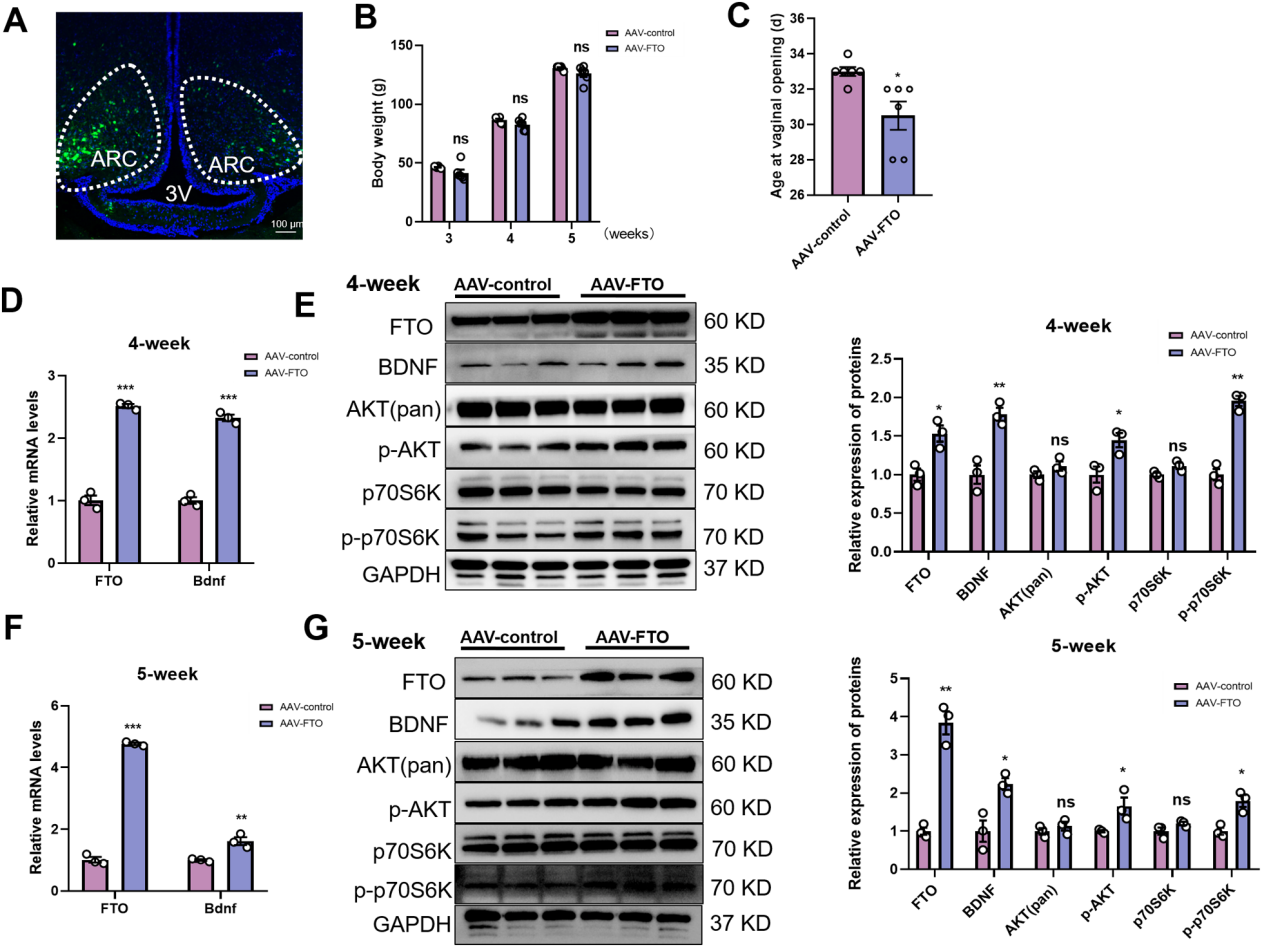
FTO overexpression in the ARC activates BDNF signaling in female rats, accelerating puberty onset. **(A)** Transduction of ARC cells with scAAV in female rats. Green, scAAV-infected cells; blue, DAPI-stained cell nuclei in the hypothalamus; dotted line, ARC; 3V, third ventricle. Scale bars, 100 μm. **(B)** The body weights of female rats at three different developmental stages did not differ between the two groups (n=6). **(C)** Vaginal opening was significantly accelerated in the AAV-FTO group relative to the AAV-control group (n=6). **(D, F)** The mRNA levels of FTO and Bdnf in the hypothalamus were determined using qRT–PCR at 4 weeks **(D)** and 5 weeks **(F)** of age (n=3). β-Actin was used as the internal reference. **(E)** Quantification of the data indicated that the protein levels of FTO, BDNF, p-AKT, and p-p70S6K were significantly increased in the AAV-FTO group compared with the control group, whereas total Akt and p70S6K levels did not change in 4-week-old female rats (n=3). **(G)** Western blot analysis indicated that the protein levels of FTO, BDNF, p-AKT, and p-p70S6K were significantly increased in the AAV-FTO groups compared with the control group, whereas total Akt and p70S6K levels did not change in 5-week-old female rats (n=3). AAV-FTO was injected into the FTO-overexpressing group, and AAV-control was injected into the AAV negative control group. ARC, arcuate nucleus; Three V, third ventricle. The results are shown as the means ± S.E.M.s. *P < 0.05, **P < 0.01, ***P < 0.001, and ns, *P* > 0.05. Two-tailed Student’s t test was used.

In this study, using self-complementing adeno-associated virus (scAAV) as a genetic engineering method in female rats, we further investigated the relationship between FTO and BDNF signaling *in vivo*. Using the Rat Brain Atlas (Paxinos and Watson, Fifth Edition) and prior localization data (25), we administered the scAAV vector into the hypothalamic ARC of rats at 3 weeks of age through precise stereotactic microinjection. As a benchmark, control animals were treated with a scAAV vector that contained enhanced green fluorescent protein (GFP). GFP was utilized to determine whether the scAAV had successfully reached the intended location (**Figure 5A**). No significant differences in body weight were observed (**Figure 5B**). Confirming FTO ARC expression as a key component for sexual development, the average time of vaginal opening in the AAV-FTO group was significantly advanced compared with that of the AAV-control group (**Figure 5C**). Hypothalamic tissues were obtained from rats aged 4 and 5 weeks for the subsequent quantification of mRNA and protein levels to determine whether the increased presence of FTO within the ARC resulted in the expected changes in genetic activity. Importantly, a significant increase in FTO expression was observed in the AAV-FTO group (**Figure 5D**). Consistent with the findings observed *in vitro*, the female rats in the AAV-FTO group exhibited markedly upregulated Bdnf mRNA expression at both 4 and 5 weeks of age (**Figures 5D, F**). Overexpression of FTO within the ARC resulted in the significant activation of BDNF and PI3K/Akt signaling at 4 weeks (**Figure 5E**). Similar changes were observed at 5 weeks of age (**Figure 5G**). Compared with those in the control group, the levels of FTO, BDNF, p-Akt, and p-p70S6K proteins in the AAV-FTO group were significantly increased at both 4 and 5 weeks. However, the levels of the total Akt and p70S6K proteins did not differ significantly among the groups. We found that no obvious change in the expression of the Kiss1, NKB, and Dyn mRNAs was observed when FTO expression increased in the ARC at 4 weeks and 5 weeks (**Supplementary Figures S3A, C**). We also measured the expression of CREB. The results showed that the levels of the CREB, p-CREB, ERK, p-ERK, and TrkB proteins were not significantly different at 4 weeks (**Supplementary Figure S3B**). However, the levels of the CREB and ERK proteins were significantly decreased and p-CREB levels were significantly increased in the AAV-FTO groups at 5 weeks. No significant differences in the levels of the p-ERK, and TrkB proteins were observed among the groups (**Supplementary Figure S3D**). These results indicated that BDNF binds to TrkB, leading to the activation of p-CREB through the PI3K/AKT signaling pathway following FTO overexpression. Consequently, the onset of pubertal development in female rats is stimulated by a prepubertal increase in FTO expression in the ARC, which activates BDNF signaling.

### Loss of FTO expression leads to delayed puberty and the inhibition of the BDNF/PI3K/Akt pathway

**Figure 6:**
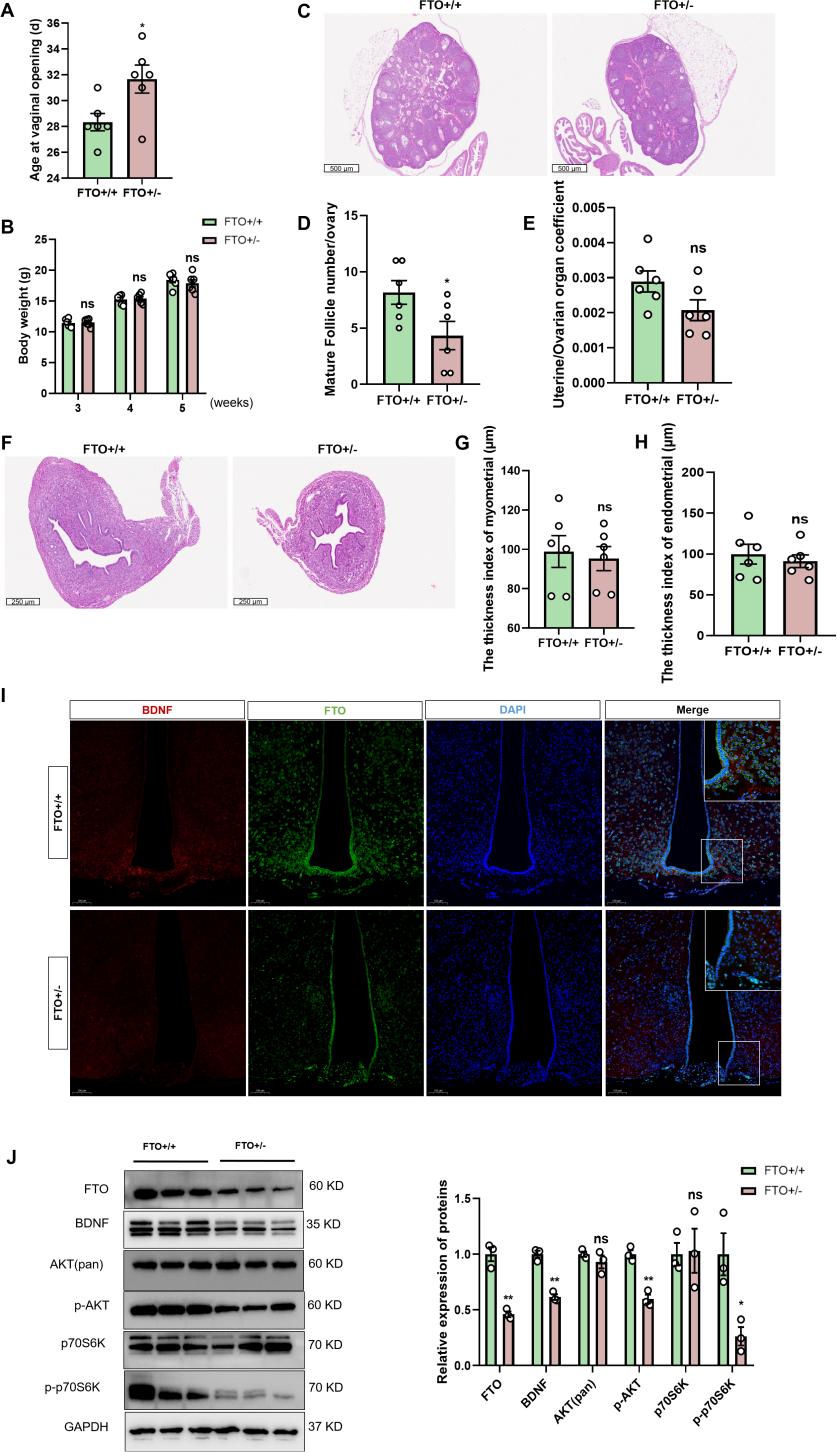
Loss of FTO expression leads to a delay in puberty and inhibition of the BDNF/PI3K/Akt pathway. **(A)** The onset of vaginal opening was significantly delayed in the FTO+/- group compared with that in the FTO+/+ group (n=6). **(B)** The body weights of female mice at different developmental stages did not differ between the two groups (n=6). **(C)** H&E staining was performed to evaluate ovarian size and follicle morphology in FTO-deficient female mice at 4 weeks (n=6; scale bar=500 μm). **(D)** Numbers of mature follicles in the ovaries of FTO+/+ and FTO+/- female mice (n=6). **(E)** The uterine/ovarian organ coefficients of FTO-deficient mice tended to decrease at 4 weeks (n=6). **(F–H)** Pathological assessment of the uterus in FTO-deficient female mice at 4 weeks of age by H&E staining **(F)**; the differences in the endometrial thickness **(G)** and myometrial thickness **(H)** between the groups were not significant (n=6). **(I)** Immunofluorescence (IF) staining for BDNF (red) and FTO (green) and nuclear staining (DAPI, blue) were significantly reduced when FTO expression was lost (n=3; scale bar=50 μm, 20 μm). **(J)** The protein levels of FTO, BDNF, p-AKT, p-p70S6K, total Akt and p70S6K in the hypothalamus of 4-week-old female mice were measured by Western blotting (n=3). The results are shown as the means ± S.E.M.s. *P < 0.05, **P < 0.01, and ns, *P* > 0.05. Two-tailed Student’s t test was used.

In this study, using FTO heterozygous knockout mice, we further investigated the relationship between BDNF signaling and FTO *in vivo*. We generated FTO heterozygous knockout (FTO+/-) mice and assessed their progression through puberty to comprehensively investigate the role of FTO in the BDNF/pi3k/Akt signaling pathway *in vivo*. FTO heterozygous knockout mice exhibited a delay in the onset of puberty (**Figure 6A**). Despite being heterozygous, the body weights of these mice remained comparable to those of their FTO+/+ counterparts (**Figure 6B**). Considering that all the female mice exhibited vaginal opening at 5 weeks of age, we subsequently utilized 4-week-old female mice as the primary experimental subjects. We further investigated the effects of FTO deficiency on gonadal tissues and adipose tissue and confirmed that the FTO+/- mice displayed a reduced ovarian size and a lower number of mature follicles compared to the control mice (**Figures 6C, D**). Decreases in the uterine and ovarian organ coefficients were observed in the FTO+/- mice at 4 weeks, but these decreases did not reach statistical significance (*p* = 0.08) (**Figure 6E**). We employed H&E staining to examine uterine structure and function. Compared with those of the FTO+/+ mice, the uteruses of the FTO+/- mice were smaller (**Figure 6F**). However, no significant differences were noted in either the endometrial thickness or the myometrial thickness at 4 weeks (**Figures 6G, H**). H&E staining also revealed a marked reduction in the diameter of fat cells and average area of adipocytes in the FTO+/- mice (**Supplementary Figures S4A, B, D**). And the fat cell density increased in the FTO+/- mice (**Supplementary Figure S4C**). We further confirmed that FTO positively regulates BDNF expression by collecting the whole brains of FTO+/- and FTO+/+ mice at 4 weeks of age. IF staining of brain sections was conducted to assess the expression of FTO and BDNF. BDNF expression was observed in the ARC of the hypothalamus, and BDNF was expressed adjacent to GnRH-positive fibers and terminals. Notably, BDNF levels were significantly decreased following the inhibition of FTO expression in the hypothalamus (**Figure 6I**). Pubertal onset (defined by vaginal opening) was significantly delayed in the FTO+/- mice (31.6 ± 1.0 days) compared with that in the control mice (28.3 ± 0.6 days, *p* < 0.05). We also analyzed the levels of proteins associated with the FTO/BDNF/PI3K/Akt signaling pathway in FTO+/+ and FTO+/- mice at 4 weeks. Hypothalamic tissue was collected for the quantification of mRNA and protein levels. GnRH mRNA expression decreased significantly in FTO-deficient mice (**Supplementary Figure S4E**). However, the levels of the Kiss1, NKB, and Dyn mRNAs did not change significantly (**Supplementary Figure S4F**). Western blot analysis confirmed the absence of FTO expression in the hypothalamic tissue of FTO-deficient mice. We observed significant decreases in the BDNF, p-Akt, and p-p70S6K protein levels following FTO deficiency, whereas total Akt and total p70S6K levels did not differ significantly at 4 weeks (**Figure 6J**). We also measured the expression of CREB. The results showed that the level of the CREB protein was significantly increased and that the level of p-CREB was significantly decreased in FTO-deficient mice at 4 weeks. Significant differences in the levels of the ERK, p-ERK, and TrkB proteins were not observed in FTO-deficient mice at 4 weeks (**Supplementary Figure S4G**). Thus, the loss of FTO expression led to delayed puberty and impaired gonadal development through the BDNF/PI3K/Akt signaling pathway.

### BDNF activates the HPG axis

**Figure 7:**
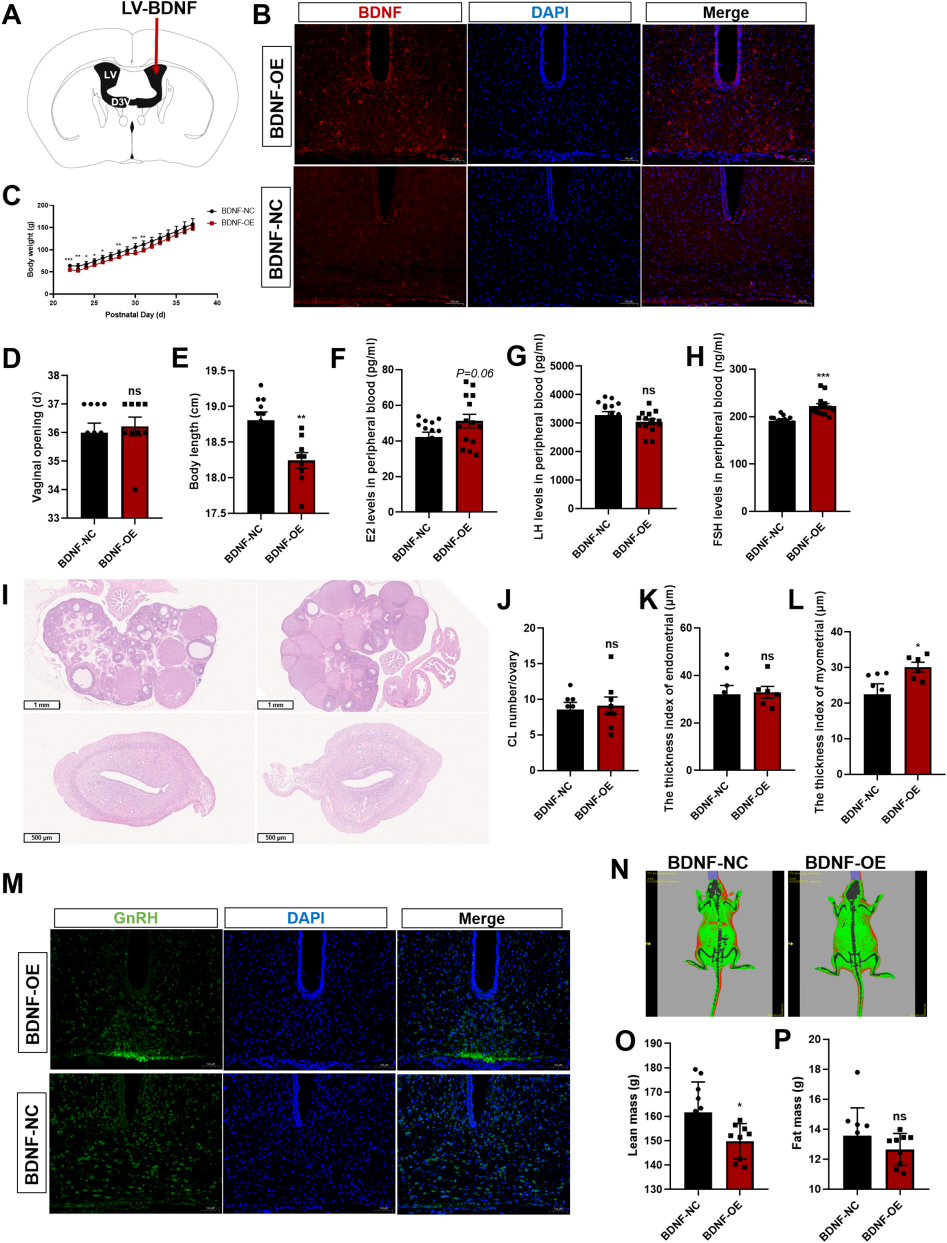
BDNF activates the HPG axis. **(A)** Schematic diagram of lentivirus (LV) microinjection into the lateral ventricle. **(B)** Immunofluorescence (IF) staining for BDNF (red) and cell nuclei (DAPI, blue) was significantly increased when BDNF was overexpressed (n=3; scale bar=100 μm). **(C)** Body weights of female rats that received LV injections beginning at postnatal week 3 (n=9–10). **(D)** The onset of vaginal opening did not differ between the BDNF-OE group and the BDNF-NC group (n=9–10). **(E)** Body length was significantly decreased in the BDNF-OE group compared with the BDNF-NC group (n=9–10). **(F–H)** Serum E2 **(F)**, LH **(G)**, and FSH **(H)** levels were determined using ELISAs in the LV-injected group (n=9–10). **(I)** H&E staining was performed to evaluate pathological changes in the ovaries and uterus of female rats after BDNF overexpression (n = 6). Scale bars=1 mm; 500 μm. **(J)** The number of corpora lutea (CLs) was determined (n = 8). **(K, L)** Pathological assessment of the uterus by H&E staining in the LV-transfected group and determination of the differences in the endometrial thickness **(K)** and myometrial thickness **(L)** between the groups. **(M)** IF staining for GnRH (green) and nuclear staining (DAPI, blue) were significantly increased when BDNF was overexpressed (n=3; scale bar=100 μm). **(N)** The body fat composition of the rats was analyzed using a small animal *in vivo* imaging system. **(O, P)** Lean mass **(O)** and fat mass **(P)** were determined using a small animal *in vivo* imaging system in the LV-transfected group (n=9–10). The results are shown as the means ± S.E.M.s. *P < 0.05, **P < 0.01, ***P < 0.001, and ns, *P* > 0.05. Two-tailed Student’s t test was used.

In this study, we further investigated the role of central BDNF in the regulation of puberty onset and reproductive function in prepubertal female Sprague–Dawley rats. We performed ICV injections of lentiviral vectors overexpressing BDNF (BDNF-OE) or a negative control (NC) (**Figure 7A**). IF staining of brain sections collected after virus injection revealed increased BDNF protein levels in the hypothalamus in the BDNF-OE group compared with those in the control group (**Figure 7B**), confirming the successful overexpression of the target gene. Following viral delivery, we monitored pubertal developmental markers daily. Female BDNF-OE rats demonstrated a markedly increased rate of body weight gain (**Figure 7C**). While a significant difference in the time of vaginal opening was not observed, the body length of the BDNF-OE female rats was significantly decreased (**Figures 7D, E**). Once all female rats achieved VO, they were euthanized for serum and tissue collection. Measurement of serum hormone levels by ELISA indicated profound endocrine alterations. Compared with the negative control group, the BDNF-OE group displayed increased concentrations of follicle-stimulating hormone (FSH) and 17β-estradiol (E2), while the concentration of luteinizing hormone (LH) did not change significantly (**Figures 7F–H**). Histological examination of ovarian and uterine sections by H&E staining revealed no significant difference in the number of CLs in the BDNF-OE group (**Figures 7I, J**). Furthermore, uterine morphological analysis revealed no significant difference in endometrial thickness indices (**Figure 7K**). However, a significant increase in the myometrial thickness was detected (**Figure 7L**). Given that GnRH is the upstream regulator of the HPG axis, we performed IF staining for GnRH in the hypothalamus. The results showed a higher density of GnRH immunofluorescence in the ARC of BDNF-OE female rats (**Figure 7M**), suggesting that central BDNF overexpression increased the expression of GnRH. We also analyzed body composition *in vivo* at the endpoint (**Figure 7N**). The results revealed a significant reduction in the whole-body lean mass of BDNF-OE rats compared with controls (**Figure 7O**), highlighting the concomitant metabolic effect of central BDNF manipulation. However, no significant difference in fat mass was observed between the groups (**Figure 7P**). Therefore, ICV-mediated BDNF overexpression efficiently activated the HPG axis, and promoted uterine development, likely through the potentiation of hypothalamic GnRH neuronal activity.

## Discussion

**Figure 8:**
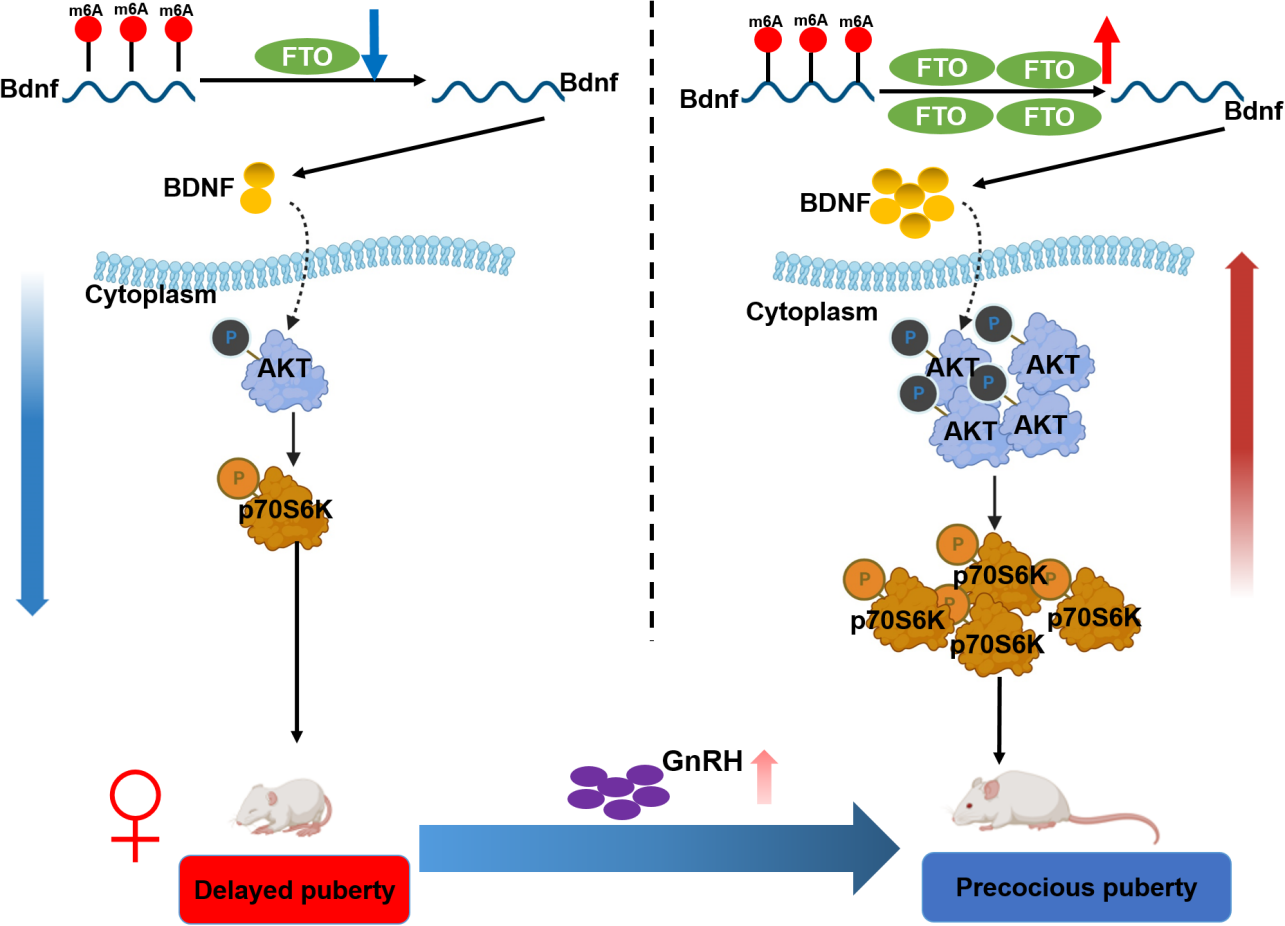
Schematic illustration summarizing the findings of the current work. Hypothalamic FTO and BDNF expression increased with pubertal development, whereas m6A methylation levels decreased. BDNF colocalized with GnRH in the arcuate nucleus (ARC). Epitranscriptomic sequencing of the ARC of female rats at various stages of normal development revealed that BDNF is a critical target of FTO-mediated modification. The overexpression of FTO increased BDNF expression by reducing the m6A methylation of Bdnf mRNA, thereby activating the BDNF/PI3K/Akt signaling pathway. Conversely, forced knockdown of BDNF expression reversed these changes. Moreover, the specific upregulation of FTO expression in the ARC prior to puberty led to accelerated pubertal development through stimulation of the BDNF/PI3K/Akt signaling pathway. In contrast, the inactivation of FTO resulted in delayed puberty and impaired gonadal development through inhibition of the BDNF/PI3K/Akt signaling pathway.

In this study, we observed that hypothalamic FTO and BDNF levels increased with pubertal development, whereas m6A methylation decreased. Epitranscriptomic sequencing of the ARC revealed that Bdnf is a critical target that undergoes FTO-mediated m6A methylation. Notably, BDNF in the ARC was expressed adjacent to GnRH-positive fibers and terminals. FTO overexpression increased BDNF expression through m6A methylation. Furthermore, FTO overexpression activated the BDNF/PI3K/Akt signaling pathway. Conversely, BDNF knockdown reversed these effects. Additionally, the targeted overexpression of FTO in the ARC accelerated puberty onset (30.5 ± 0.8 days vs. 33 ± 0.3 days in control animals, *p* < 0.05) by activating the BDNF/PI3K/Akt pathway. In contrast, the inactivation of FTO resulted in delayed puberty (31.6 ± 1.0 days vs. 28.3 ± 0.6 days in control animals; *p* < 0.05) and inhibited the BDNF/PI3K/Akt signaling pathway. Central BDNF overexpression efficiently activated the HPG axis (**Figure 8**). These findings suggest that hypothalamic FTO plays a critical role in the progression of puberty through the activation of the BDNF/PI3K/Akt signaling pathway.

Epigenetic modifications play important roles in regulating pubertal development (26–28). m6A methylation is enriched in the hypothalamus and its level decreases with pubertal development (29). FTO is a prevalent RNA demethylase that is abundantly expressed in the brain, particularly within the hypothalamus (30). The expression of FTO is associated with pubertal development, and its role in regulating calcium ion signaling in the brain has been well established (20). Moreover, FTO plays a crucial role in modulating hippocampal activity through the regulation of BDNF expression (17).A recent study indicated that hippocampal FTO participates in novel object recognition (NOR) memory reconsolidation through BDNF–TrkB signaling (31). These findings suggest that BDNF could be an important molecular target of FTO. Notably, our joint analysis revealed that the m6A methylation of Bdnf mRNA in the hypothalamus of female rats significantly decreased during puberty onset. However, the m6A methylation of Bdnf mRNA decreased. Collectively, these results indicate that BDNF may be a target of the demethylase FTO in the hypothalamus.

FTO overexpression increased BDNF expression through m6A methylation. The results of functional experiments using GT1–7 cells suggested that a significant increase in FTO expression led to only very minor changes in most endpoint indicators, while a reduction in FTO expression had a much greater effect on various indicators. Regarding the reason for this imbalance, we speculated that on the one hand, the signaling pathways and biological processes regulated by FTO might have an inherent saturation point. Namely, a moderate, physiological level of FTO may be sufficient to fully activate these pathways. On the other hand, the massive overexpression of FTO might trigger compensatory feedback mechanisms within the cell to buffer its effects and maintain metabolic and transcriptional homeostasis. Negative regulatory factors may be upregulated or adaptive stress responses may be initiated, thereby weakening the overall effect. In contrast, reducing FTO expression might disable a key regulatory node, shifting the cell from its compensatory range and thus producing more dramatic phenotypic consequences. It should be emphasized that although FTO-mediated upregulation of Bdnf was observed in GT1–7 cells, endogenous, terminally differentiated GnRH neurons have been shown not to express BDNF (16). In contrast, BDNF is primarily supplied to GnRH neurons via a paracrine mechanism by neighboring cells in the hypothalamic environment. Therefore, the increased Bdnf expression detected in this immortalized cell line likely reflects its transformed, embryonic origin-specific characteristics rather than a physiologically relevant mechanism in mature GnRH neurons. The significance of this finding in GT1–7 cells does not lie in reproducing the autocrine loop in native neurons, but in demonstrating that FTO has the intrinsic ability to regulate the BDNF within a neuronal context expressing the necessary transcriptional machinery. The main site of action of FTO *in vivo* is likely to be in those afferent neurons that do express BDNF and subsequently influence GnRH neuron activity.

Our findings suggest that FTO is a key regulator of the timing of puberty onset. The observation that puberty eventually occurred in all groups indicated that FTO is not an absolute prerequisite for the activation of the HPG axis. Instead, its expression level was likely to fine-tune the timing of this process by modulating the metabolic information integrated in the hypothalamus and influencing the GnRH pulse generator. Our research focused on the central role of FTO in the ARC of the hypothalamus. However, FTO is also expressed in peripheral metabolic tissues, including the gonads (18). Therefore, although our data suggested that central FTO overexpression is sufficient to increase the activation of the HPG axis, we cannot rule out the possibility of a direct effect of FTO on the ovaries and testes. The observed changes in the gonads might be the result of the combined actions of central drive and local direct effects. Future studies using tissue-specific (e.g., gonad-specific) FTO knockout or overexpression models will be crucial for clarifying the exact contributions of central and peripheral FTO to the timing of puberty onset.

BDNF, which is produced by both neurons and glial cells in the brain, is the most prominent and widely and complexly distributed neurotrophic factor in the mammalian central nervous system. Our data indicated that BDNF in the ARC was expressed adjacent to GnRH-positive fibers and terminals. Recombinant BDNF protein stimulation in E12.5 mouse olfactory tissue culture promoted the growth of GnRH axons by activating the intracellular Ca^2+^–CREB signaling pathway (9). A clinical study of functional hypothalamic amenorrhea (FHA) revealed that the plasma BDNF concentration in FHA patients was significantly lower than that in healthy controls (32). BDNF can bind to TrkB receptors located on the surface of GnRH neurons and activate the TrkB/Ca^2+^/CREB signaling pathway (33). p-CREB further activates the transcription factor c-FOS and promotes the transcription of GnRH (34). Female rats in which FTO was overexpressed in the ARC exhibited advanced puberty. Moreover, we demonstrated that a reduction in FTO expression in female mice resulted in delayed puberty, which was mediated by BDNF signaling. The absence of FTO leads to the dysregulation of the expression of specific genes, such as *Bdnf*, along with their associated signaling pathways. Therefore, FTO may regulate pubertal development through BDNF signaling.

The neurotrophic and prosurvival effects of BDNF are mediated mainly by the activation of TrkB receptors located on the cell surface. TrkB serves as an intrinsic receptor for BDNF. The synergistic action of BDNF and TrkB initiates various intracellular signaling pathways, particularly the PI3K/Akt signaling pathway (35, 36). PI3K/Akt is a representative antiapoptotic signaling pathway (37). Research has indicated that PI3K signaling plays an important role in modulating reproductive function within the hypothalamus. PI3K signaling is vital for controlling fertility and the release of GnRH. It can directly affect important neuropeptides expression (38). PI3K signaling could increase CREB activation, promoting GnRH expression. Our research also revealed that the m6A methylation of BDNF by FTO influences the expression of GnRH at the onset of puberty via PI3K/Akt/CREB signaling.

Exogenous BDNF can upregulate the expression of the Kiss1, Tac2, and Gnrh1 genes simultaneously via the ERK/CREB signaling pathway and modulate the secretion of luteinizing hormone (LH) and follicle-stimulating hormone (FSH) from pituitary cells by influencing the expression of the GnRH and KNDy genes. In this manner, BDNF participates in the regulation of central gonadotropin axis activity in female sheep (16). We observed a clear increase in BDNF overexpression, as evidenced by increased hypothalamic GnRH levels and elevated peripheral FSH and E2 levels; these changes did not translate to a statistically significant acceleration of VO. These findings suggest that BDNF plays a more direct role in facilitating puberty onset. However, our data align with the complex, multifaceted nature of BDNF signaling. It is plausible that the specific timing, location, and magnitude of BDNF overexpression in our model was sufficient to stimulate GnRH synthesis and partial gonadotropin release (FSH) but was insufficient to trigger the full sequence of events required to significantly advance the first estrous cycle marked by VO. Concurrently, BDNF-OE induced profound metabolic alterations, repartitioning energy resources away from linear growth and toward systems supporting a reproductive state. The significant reduction in body length and lean mass, coupled with accelerated weight gain, indicates a BDNF-driven shift in energy allocation. This finding is consistent with the well-established role of BDNF in the hypothalamus in the regulation of appetite and energy metabolism, which may also play a role in the central neuroendocrine network that controls the function of the HPGA, thereby influencing the progression of puberty. Our results suggest that central BDNF signaling may prioritize metabolic fuel for processes immediately beneficial for reproduction at the expense of somatic growth. This process represents a fascinating physiological trade-off orchestrated by the brain, aligning metabolic status with reproductive competence.

Each model used in this study was selected to address a specific issue within a logical workflow. The rat model enabled us to establish the *in vivo* physiological correlation between peripheral BDNF levels, central FTO expression, and the timing of puberty onset in a complete system. The mouse genetic model provided causal evidence for the role of endogenous FTO. The GT1–7 cell line was used because of its practicality for conducting rigorous gain-of-function and loss-of-function experiments as well as detailed analyses of signaling pathways. Based on the results of this study, the main site of action of BDNF in our *in vivo* models may not have been the GnRH neuron cell body itself but rather its termini in the median eminence or, more likely, upstream afferent neurons that respond to BDNF and subsequently regulate GnRH release. This result can explain the observed physiological effects without the need for a direct effect of BDNF on the postnatal GnRH cell body. We focused on a central FTO–BDNF pathway that ultimately affects GnRH release, and its mechanism may involve both direct (acting on terminals) and indirect (acting on afferent input) effects.

However, it should be noted that we were limited to using GT1–7 cells for the *in vitro* experiments. GT1–7 cells are valuable *in vitro* tools for mechanistic studies. Their embryonic origin and immortalized nature imply that their responses may not fully replicate those of mature, terminally differentiated GnRH neurons *in vivo* (9). Due to the lack of individual-level data, a correlation analysis could not be conducted between the FTO levels in animals and the onset time of puberty. Another primary limitation of this study was the use of global ICV injection for the overexpression of BDNF. While effective at inducing the overexpression of BDNF within the hypothalamus, this method did not allow us to pinpoint the exact nuclei responsible for the observed effects. The distinct outcomes—strong GnRH and FSH responses without accelerated VO—may have been due to the simultaneous activation of both stimulatory and inhibitory pathways within different hypothalamic regions. Future studies employing Cre-lox technology for cell-specific and nucleus-specific BDNF manipulation will be crucial for dissecting these complex neural circuits. The small sample size is a key limitation of our study. Future research in larger cohorts is crucial for confirming and expanding these preliminary observations.

In summary, our research highlights the critical role of BDNF in regulating puberty onset. Our methodology integrated m6A profiling and gene expression analysis, which facilitated the identification of key mRNAs modified by FTO. Among the transcripts identified, those exhibiting alterations in m6A methylation were found to be significantly involved in the BDNF pathway. Notably, these changes—particularly in genes associated with the BDNF pathway—may accelerate puberty onset associated with FTO overexpression. The FTO-mediated m6A methylation of BDNF regulates the PI3K/Akt pathway in the hypothalamus, thereby influencing puberty onset.

## Data Availability

The datasets presented in this study can be found in online repositories. The names of the repository/repositories and accession number(s) can be found here: https://www.ncbi.nlm.nih.gov/geo/, GSE253114.

## Supplementary material

Supplementary Figure 1 Enrichment analyses of differentially expressed genes in the ARCs of 21-day-old and 35-day-old female rats. **(A)** Colorimetric quantification of reduced m6A methylation levels in the hypothalamus during puberty (n=5). **(B)** The mRNA expression of FTO in the hypothalamus increased significantly with puberty onset, as shown by qPCR (n=5). **(C)** The protein expression of FTO in the hypothalamus increased significantly with puberty onset, as shown by Western blotting (n=3). **(D)** Pie charts showing the distribution of m6A peaks in different RNA regions (CDS, 5’ UTR, 3’ UTR, start codon, and stop codon) in the ARCs of 21-day-old and 35-day-old female rats. **(E)** The distributions of genes with 1, 2, 3, 4, 5, and 6 or more m6A peaks in the ARCs of 21-day-old and 35-day-old female rats. **(F)** Gene Ontology (GO) enrichment analysis of the differentially expressed genes in the ARC of 35-day-old female rats based on RNA-seq data. **(G)** Kyoto Encyclopedia of Genes and Genomes (KEGG) pathway analysis of differentially expressed genes in the ARC of 35-day-old female rats based on RNA-seq data. ARC, arcuate nucleus. Pathway analysis was performed using the KEGG database (http://www.kegg.jp/kegg/kegg1.html).

Supplementary Figure 2 Three Bdnf-silencing plasmids were successfully constructed. **(A)** The expression of Bdnf mRNA in control and cells transfected with the three Bdnf shRNAs was determined by qPCR (n=3). **(B)** BDNF protein levels were determined by Western blotting (n=3). The bars represent the means ± S.E.M.s. **P* < 0.05, ***P* < 0.01, ****P* < 0.001, and ns, *P* > 0.05 compared with the shRNA-control group according to Student’s t test.

Supplementary Figure 3 Overexpression of FTO in the ARC of the hypothalamus in female mice activates CREB signaling but has no effect on KNDy neurons. **(A)** The mRNA levels of Kiss1, NKB, and Dyn in the hypothalamus were determined using qRT–PCR at 4 weeks **(A)** and 5 weeks **(C)** (n=3). β-Actin was used as the internal reference. **(B)** Quantification of the data indicated that the protein levels of CREB, p-CREB, ERK, p-ERK, and TrkB did not differ significantly among the groups at 4 weeks (n=3). **(D)** Western blot analysis indicated that the levels of the CREB and ERK proteins were significantly decreased and p-CREB levels were significantly increased in the AAV-FTO groups at 5 weeks. No significant differences in the levels of the p-ERK and TrkB proteins were observed among the groups (n=3). AAV-FTO was injected into the FTO-overexpressing group, and AAV-control was injected into the AAV negative control group. ARC, arcuate nucleus; Three V, third ventricle. The results are shown as the means ± S.E.M.s. **P* < 0.05, ***P* < 0.01, ****P* < 0.001, and ns, *P* > 0.05. Two-tailed Student’s t test was used.

Supplementary Figure 4 FTO deficiency in female mice inhibits the CREB signaling but has no effect on KNDy neurons. **(A)** H&E staining was used to evaluate pathological changes in white fat from female mice at 4 weeks after FTO knockout. Scale bars=100 μm. **(B)** The diameter of fat cells decreased in the FTO+/- mice at 4 weeks (n=3). **(C)** The fat cell density increased in the FTO+/- mice (n=3). **(D)** The average area of adipocytes decreased in the FTO+/- mice (n=3). **(E)** The mRNA level of GnRH in the hypothalamus of FTO-deficient mice was decreased at 4 weeks (n=3). **(F)** No significant changes in the mRNA levels of Kiss1, NKB, or Dyn were observed in the hypothalamus of 4-week-old FTO-deficient mice (n=3). β-Actin was used as the internal reference. **(G)** Western blot analysis showed that the level of the CREB protein was significantly increased, and p-CREB levels were significantly decreased in 4-week-old FTO-deficient mice. No significant differences in the levels of the ERK, p-ERK, or TrkB protein were observed in 4-week-old FTO-deficient mice (n=3). The results are shown as the means ± S.E.M.s. **P* < 0.05, and ns, *P* > 0.05. Two-tailed Student’s t test was used.

## References

[1] Latronico AC Brito VN Carel JC. Causes, diagnosis, and treatment of central precocious puberty. Lancet Diabetes Endocrinol. (2016) 4:265–74. doi: 10.1016/S2213-8587(15)00380-0, PMID: 26852255

[2] Klein DA Emerick JE Sylvester JE Vogt KS. Disorders of puberty: an approach to diagnosis and management. Am Fam Physician. (2017) 96:590–9., PMID: 29094880

[3] Yoo JH. Effects of early menarche on physical and psychosocial health problems in adolescent girls and adult women. Korean J Pediatr. (2016) 59:355–61. doi: 10.3345/kjp.2016.59.9.355, PMID: 27721839 PMC5052133

[4] Dees W Hiney J Srivastava V. IGF-1 influences gonadotropin-releasing hormone regulation of puberty. Neuroendocrinology. (2021) 111:1151–63. doi: 10.1159/000514217, PMID: 33406521 PMC8257778

[5] Dorfman M Garcia-Rudaz C Alderman Z Kerr B Lomniczi A Dissen G. Loss of Ntrk2/Kiss1r signaling in oocytes causes premature ovarian failure. Endocrinology. (2014) 155:3098–111. doi: 10.1210/en.2014-1111, PMID: 24877631 PMC4097998

[6] Hempstead BL. Dissecting the diverse actions of pro- and mature neurotrophins. Curr Alzheimer Res. (2006) 3:19–24. doi: 10.2174/156720506775697061, PMID: 16472198

[7] Li C Zhou X. The potential roles of neurotrophins in male reproduction. Reproduction. (2013) 145:R89–95. doi: 10.1530/REP-12-0466, PMID: 23404847

[8] Tapia-Arancibia L Rage F Givalois L Arancibia S. Physiology of BDNF: focus on hypothalamic function. Front Neuroendocrinol. (2004) 25:77–107. doi: 10.1016/j.yfrne.2004.04.001, PMID: 15571756

[9] Cronin AS Horan TL Spergel DJ Brooks AN Hastings MH Ebling FJ. Neurotrophic effects of BDNF on embryonic gonadotropin-releasing hormone (GnRH) neurons. Eur J Neurosci. (2004) 20:338–44. doi: 10.1111/j.1460-9568.2004.03490.x, PMID: 15233743

[10] Seifer D Feng B Shelden R Chen S Dreyfus C. Brain-derived neurotrophic factor: a novel human ovarian follicular protein. J Clin Endocrinol Metab. (2002) 87:655–9. doi: 10.1210/jcem.87.2.8213, PMID: 11836300

[11] Kawamura K Kawamura N Mulders S Sollewijn Gelpke M Hsueh A. Ovarian brain-derived neurotrophic factor (BDNF) promotes the development of oocytes into preimplantation embryos. Proc Natl Acad Sci United States America. (2005) 102:9206–11. doi: 10.1073/pnas.0502442102, PMID: 15967989 PMC1166611

[12] Kawamura K Kawamura N Fukuda J Kumagai J Hsueh A Tanaka T. Regulation of preimplantation embryo development by brain-derived neurotrophic factor. Dev Biol. (2007) 311:147–58. doi: 10.1016/j.ydbio.2007.08.026, PMID: 17880937

[13] Cordeira JW Frank L Sena-Esteves M Pothos EN Rios M. Brain-derived neurotrophic factor regulates hedonic feeding by acting on the mesolimbic dopamine system. J Neurosci. (2010) 30:2533–41. doi: 10.1523/JNEUROSCI.5768-09.2010, PMID: 20164338 PMC2846779

[14] Unger TJ Calderon GA Bradley LC Sena-Esteves M Rios M. Selective deletion of Bdnf in the ventromedial and dorsomedial hypothalamus of adult mice results in hyperphagic behavior and obesity. J Neurosci. (2007) 27:14265–74. doi: 10.1523/JNEUROSCI.3308-07.2007, PMID: 18160634 PMC6673437

[15] Xu B Goulding EH Zang K Cepoi D Cone RD Jones KR. Brain-derived neurotrophic factor regulates energy balance downstream of melanocortin-4 receptor. Nat Neurosci. (2003) 6:736–42. doi: 10.1038/nn1073, PMID: 12796784 PMC2710100

[16] Przybyl BJ Szlis M Wojcik-Gladysz A. Brain-derived neurotrophic factor (BDNF) affects the activity of the gonadotrophic axis in sheep. Horm Behav. (2021) 131:104980. doi: 10.1016/j.yhbeh.2021.104980, PMID: 33872927

[17] Spychala A Rüther U. FTO affects hippocampal function by regulation of BDNF processing. PLoS One. (2019) 14:e0211937. doi: 10.1371/journal.pone.0211937, PMID: 30730976 PMC6366932

[18] Gerken T Girard C Tung Y Webby C Saudek V Hewitson K. The obesity-associated FTO gene encodes a 2-oxoglutarate-dependent nucleic acid demethylase. Sci (New York N.Y.). (2007) 318:1469–72. doi: 10.1126/science.1151710, PMID: 17991826 PMC2668859

[19] Li Z Weng H Su R Weng X Zuo Z Li C. FTO plays an oncogenic role in acute myeloid leukemia as a N-methyladenosine RNA demethylase. Cancer Cell. (2017) 31:127–41. doi: 10.1016/j.ccell.2016.11.017, PMID: 28017614 PMC5234852

[20] Zang S Yin X Li P. FTO-mediated mA demethylation regulates GnRH expression in the hypothalamus via the PLCβ3/Ca/CAMK signalling pathway. Commun Biol. (2023) 6:1297. doi: 10.1038/s42003-023-05677-2, PMID: 38129517 PMC10739951

[21] Ip CK. An optimized protocol for establishing a chronic stress model in mice. Star Protoc. (2021) 2(2):100448. doi: 10.1016/j.xpro.2021.100448, PMID: 33912847 PMC8065287

[22] Kanehisa M Furumichi M Sato Y Matsuura Y Ishiguro-Watanabe M. KEGG: biological systems database as a model of the real world. Nucleic Acids Res. (2025) 53:D672–7. doi: 10.1093/nar/gkae909, PMID: 39417505 PMC11701520

[23] Wu R Chen Y Liu Y Zhuang L Chen W Zeng B. m6A methylation promotes white-to-beige fat transition by facilitating Hif1a translation. EMBO Rep. (2021) 22:e52348. doi: 10.15252/embr.202052348, PMID: 34569703 PMC8567275

[24] Han Z Niu T Chang J Lei X Zhao M Wang Q. Crystal structure of the FTO protein reveals basis for its substrate specificity. Nature. (2010) 464:1205–9. doi: 10.1038/nature08921, PMID: 20376003

[25] Zang S Yin X Li P. Downregulation of TTF1 in the rat hypothalamic ARC or AVPV nucleus inhibits Kiss1 and GnRH expression, leading to puberty delay. Reprod Biol Endocrinol. (2021) 19:30. doi: 10.1186/s12958-021-00710-7, PMID: 33622350 PMC7901190

[26] Argente J Dunkel L Kaiser UB Latronico AC Lomniczi A Soriano-Guillen L. Molecular basis of normal and pathological puberty: from basic mechanisms to clinical implications. Lancet Diabetes Endocrinol. (2023) 11:203–16. doi: 10.1016/S2213-8587(22)00339-4, PMID: 36620967 PMC10198266

[27] Shen Y Zhou S Zhao X Li H Sun J. Characterization of genome-wide DNA methylation and hydroxymethylation in mouse arcuate nucleus of hypothalamus during puberty process. Front Genet. (2020) 11:626536. doi: 10.3389/fgene.2020.626536, PMID: 33381157 PMC7768033

[28] Zhou S Shen Y Zang S Yin X Li P. The epigenetic role of HTR1A antagonist in facilitaing GnRH expression for pubertal initiation control. Mol Ther Nucleic Acids. (2021) 25:198–206. doi: 10.1016/j.omtn.2021.05.014, PMID: 34458005 PMC8368778

[29] Shafik A Zhang F Guo Z Dai Q Pajdzik K Li Y. N6-methyladenosine dynamics in neurodevelopment and aging, and its potential role in Alzheimer’s disease. Genome Biol. (2021) 22:17. doi: 10.1186/s13059-020-02249-z, PMID: 33402207 PMC7786910

[30] Fredriksson R Hägglund M Olszewski P Stephansson O Jacobsson J Olszewska A. The obesity gene, FTO, is of ancient origin, up-regulated during food deprivation and expressed in neurons of feeding-related nuclei of the brain. Endocrinology. (2008) 149:2062–71. doi: 10.1210/en.2007-1457, PMID: 18218688

[31] Chang R Zhu S Peng J Lang Z Zhou X Liao H. The hippocampal FTO-BDNF-TrkB pathway is required for novel object recognition memory reconsolidation in mice. Trans Psychiatry. (2023) 13:349. doi: 10.1038/s41398-023-02647-4, PMID: 37963912 PMC10645923

[32] Podfigurna-Stopa A Casarosa E Luisi M Czyzyk A Meczekalski B Genazzani A. Decreased plasma concentrations of brain-derived neurotrophic factor (BDNF) in patients with functional hypothalamic amenorrhea. Gynecological Endocrinol. (2013) 29:817–20. doi: 10.3109/09513590.2013.813472, PMID: 23844985

[33] Li J He P Zhang J Li N. Orcinol glucoside improves the depressive-like behaviors of perimenopausal depression mice through modulating activity of hypothalamic-pituitary-adrenal/ovary axis and activating BDNF- TrkB-CREB signaling pathway. Phytotherapy research: PTR. (2021) 35:5795–807. doi: 10.1002/ptr.7237, PMID: 34382261

[34] Duan W Shin J Jameson J. Estradiol suppresses phosphorylation of cyclic adenosine 3’, 5’-monophosphate response element binding protein (CREB) in the pituitary: evidence for indirect action via gonadotropin-releasing hormone. Mol Endocrinol (Baltimore Md.). (1999) 13:1338–52. doi: 10.1210/mend.13.8.0322, PMID: 10446907

[35] Yoshii A Constantine-Paton M. Postsynaptic BDNF-TrkB signaling in synapse maturation, plasticity, and disease. Dev Neurobiol. (2010) 70:304–22. doi: 10.1002/dneu.20765, PMID: 20186705 PMC2923204

[36] Yang J Yan H Li S Zhang M. Berberine ameliorates MCAO induced cerebral ischemia/reperfusion injury via activation of the BDNF-trkB-PI3K/akt signaling pathway. Neurochemical Res. (2018) 43:702–10. doi: 10.1007/s11064-018-2472-4, PMID: 29357017

[37] Li Y Xiang L Wang C Song Y Miao J Miao M. Protection against acute cerebral ischemia/reperfusion injury by Leonuri Herba Total Alkali via modulation of BDNF-TrKB-PI3K/Akt signaling pathway in rats. Biomedicine pharmacotherapy = Biomedecine pharmacotherapie. (2021) 133:111021. doi: 10.1016/j.biopha.2020.111021, PMID: 33227709

[38] Beymer M Negrón A Yu G Wu S Mayer C Lin R. Kisspeptin cell-specific PI3K signaling regulates hypothalamic kisspeptin expression and participates in the regulation of female fertility. Am J Physiol Endocrinol Metab. (2014) 307:E969–82. doi: 10.1152/ajpendo.00385.2014, PMID: 25269483 PMC4254985

