# Supplementary figures and images for: Hypothalamic FTO upregulates BDNF to promote GnRH expression through the PI3K/Akt pathway, leading to precocious puberty
Source: Front Endocrinol (Lausanne). 2025 Oct 31;16:1665391. doi: 10.3389/fendo.2025.1665391 (PMC12615161; doi:10.3389/fendo.2025.1665391)

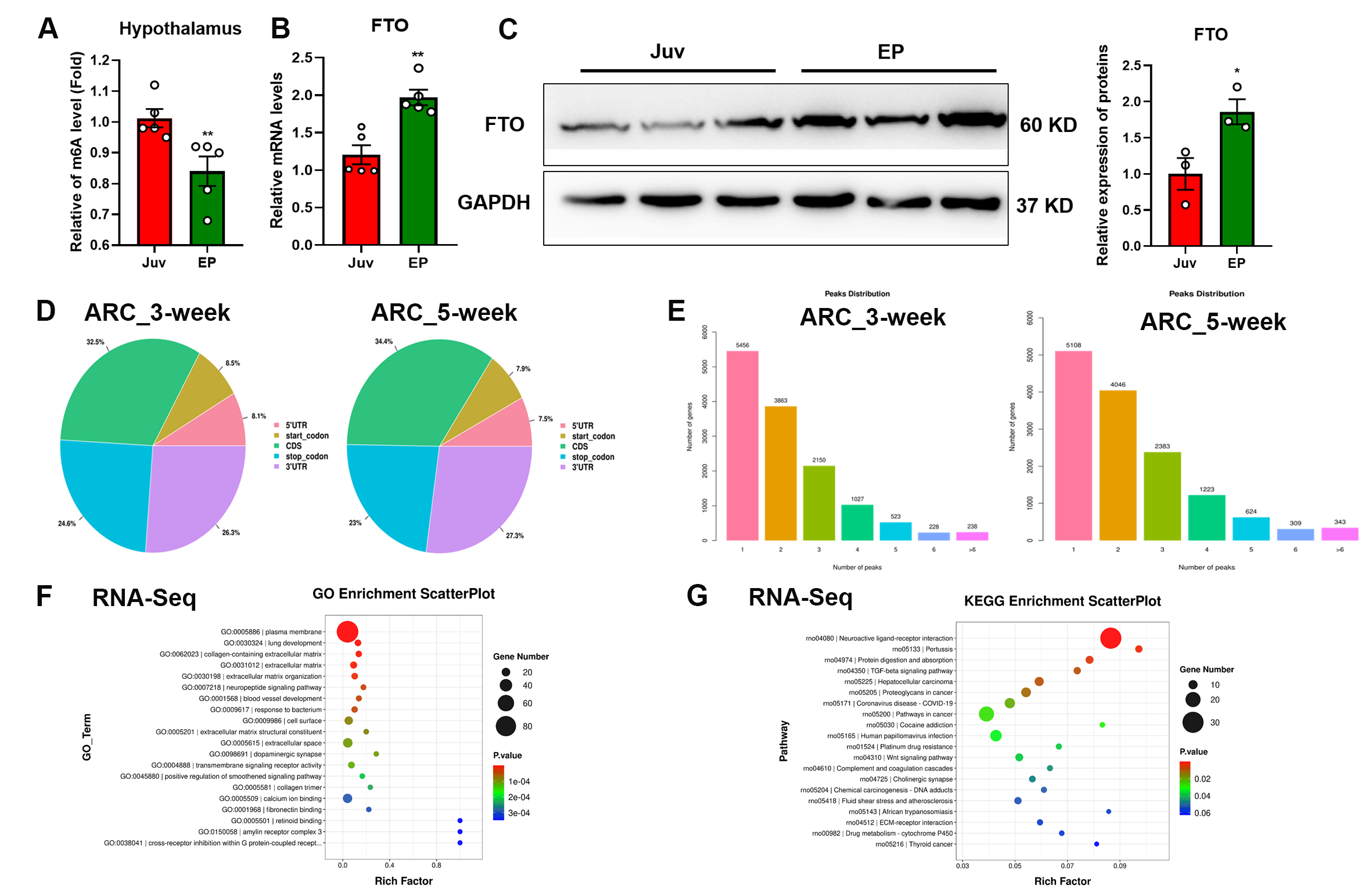

Supplement: Supplementary Figure 1 — Enrichment analyses of differentially expressed genes in the ARCs of 21-day-old and 35-day-old female rats. (A) Colorimetric quantification of reduced m6A methylation levels in the hypothalamus during puberty (n=5). (B) The mRNA expression of FTO in the hypothalamus increased significantly with puberty onset, as shown by qPCR (n=5). (C) The protein expression of FTO in the hypothalamus increased significantly with puberty onset, as shown by Western blotting (n=3). (D) Pie charts showing the distribution of m6A peaks in different RNA regions (CDS, 5’ UTR, 3’ UTR, start codon, and stop codon) in the ARCs of 21-day-old and 35-day-old female rats. (E) The distributions of genes with 1, 2, 3, 4, 5, and 6 or more m6A peaks in the ARCs of 21-day-old and 35-day-old female rats. (F) Gene Ontology (GO) enrichment analysis of the differentially expressed genes in the ARC of 35-day-old female rats based on RNA-seq data. (G) Kyoto Encyclopedia of Genes and Genomes (KEGG) pathway analysis of differentially expressed genes in the ARC of 35-day-old female rats based on RNA-seq data. ARC, arcuate nucleus. Pathway analysis was performed using the KEGG database (http://www.kegg.jp/kegg/kegg1.html). [file Image1.tif]

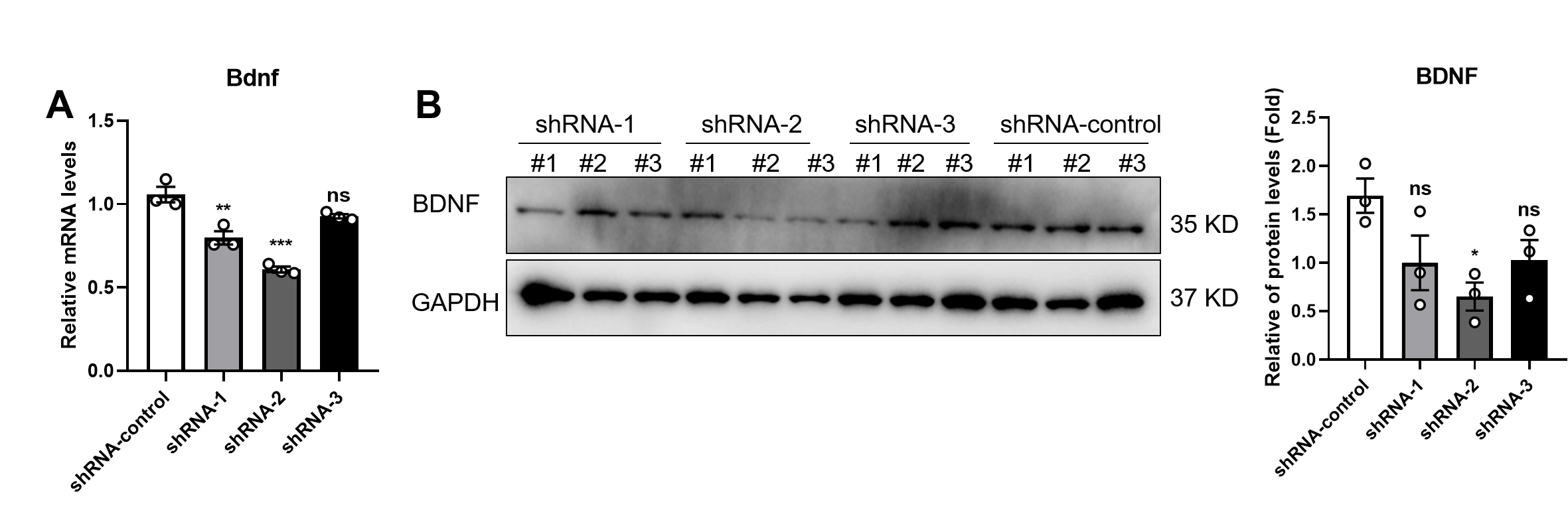

Supplement: Supplementary Figure 2 — Three Bdnf-silencing plasmids were successfully constructed. (A) The expression of Bdnf mRNA in control and cells transfected with the three Bdnf shRNAs was determined by qPCR (n=3). (B) BDNF protein levels were determined by Western blotting (n=3). The bars represent the means ± S.E.M.s. *P < 0.05, **P < 0.01, ***P < 0.001, and ns, P > 0.05 compared with the shRNA-control group according to Student’s t test. [file Image2.tif]

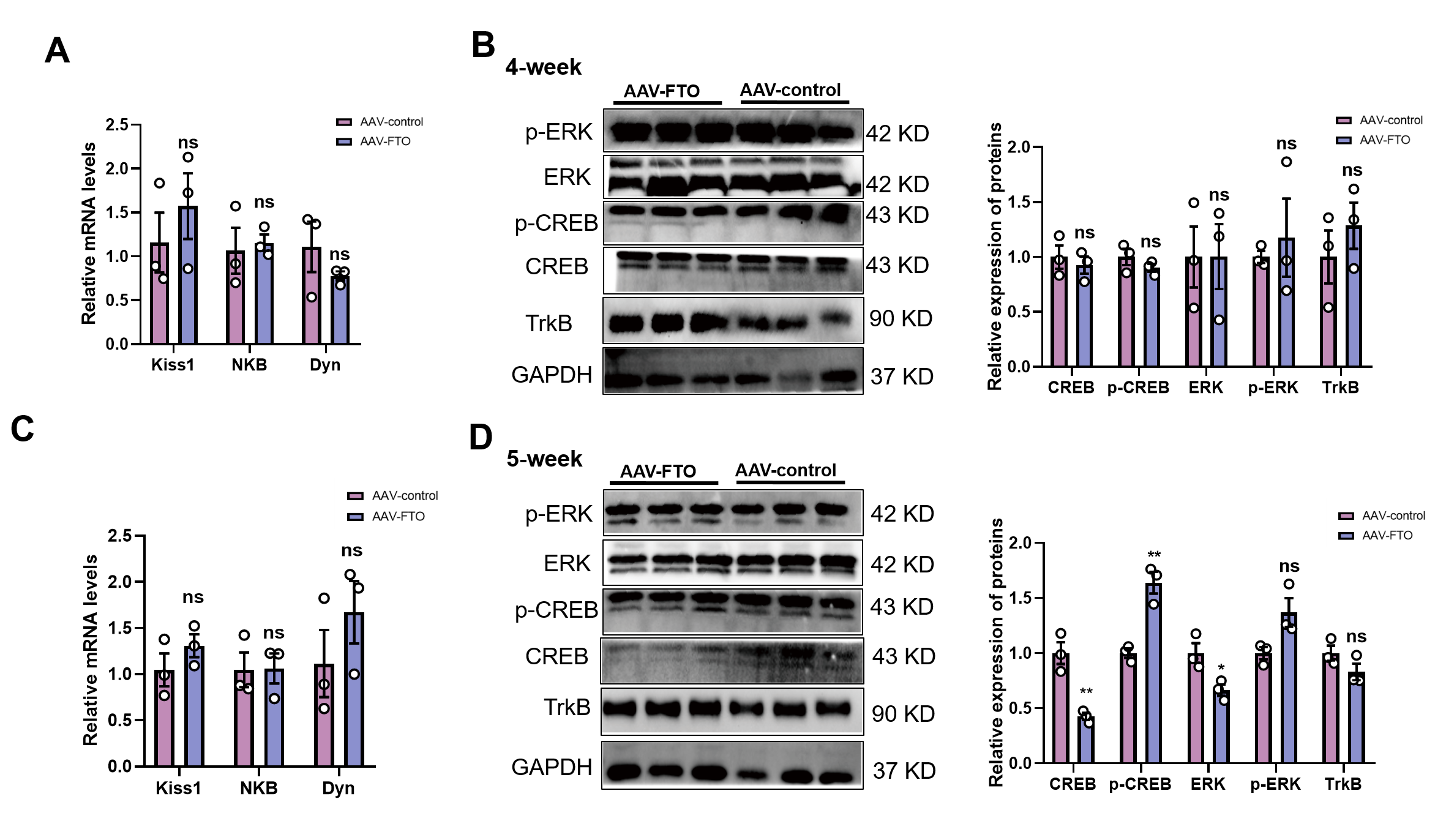

Supplement: Supplementary Figure 3 — Overexpression of FTO in the ARC of the hypothalamus in female mice activates CREB signaling but has no effect on KNDy neurons. (A) The mRNA levels of Kiss1, NKB, and Dyn in the hypothalamus were determined using qRT–PCR at 4 weeks (A) and 5 weeks (C) (n=3). β-Actin was used as the internal reference. (B) Quantification of the data indicated that the protein levels of CREB, p-CREB, ERK, p-ERK, and TrkB did not differ significantly among the groups at 4 weeks (n=3). (D) Western blot analysis indicated that the levels of the CREB and ERK proteins were significantly decreased and p-CREB levels were significantly increased in the AAV-FTO groups at 5 weeks. No significant differences in the levels of the p-ERK and TrkB proteins were observed among the groups (n=3). AAV-FTO was injected into the FTO-overexpressing group, and AAV-control was injected into the AAV negative control group. ARC, arcuate nucleus; Three V, third ventricle. The results are shown as the means ± S.E.M.s. *P < 0.05, **P < 0.01, ***P < 0.001, and ns, P > 0.05. Two-tailed Student’s t test was used. [file Image3.tif]

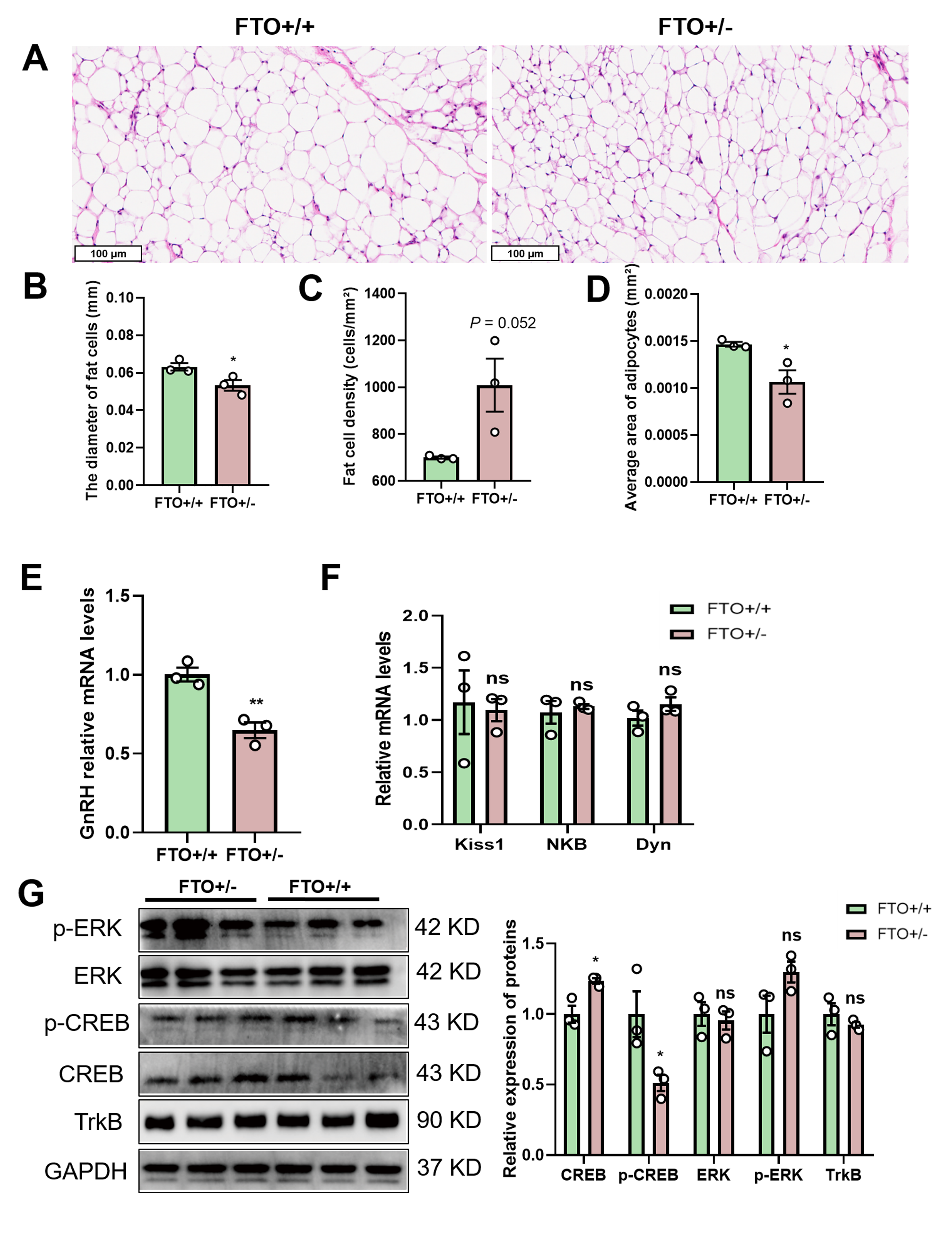

Supplement: Supplementary Figure 4 — FTO deficiency in female mice inhibits the CREB signaling but has no effect on KNDy neurons. (A) H&E staining was used to evaluate pathological changes in white fat from female mice at 4 weeks after FTO knockout. Scale bars=100 μm. (B) The diameter of fat cells decreased in the FTO+/- mice at 4 weeks (n=3). (C) The fat cell density increased in the FTO+/- mice (n=3). (D) The average area of adipocytes decreased in the FTO+/- mice (n=3). (E) The mRNA level of GnRH in the hypothalamus of FTO-deficient mice was decreased at 4 weeks (n=3). (F) No significant changes in the mRNA levels of Kiss1, NKB, or Dyn were observed in the hypothalamus of 4-week-old FTO-deficient mice (n=3). β-Actin was used as the internal reference. (G) Western blot analysis showed that the level of the CREB protein was significantly increased, and p-CREB levels were significantly decreased in 4-week-old FTO-deficient mice. No significant differences in the levels of the ERK, p-ERK, or TrkB protein were observed in 4-week-old FTO-deficient mice (n=3). The results are shown as the means ± S.E.M.s. *P < 0.05, and ns, P > 0.05. Two-tailed Student’s t test was used. [file Image4.tif]
