# Supplementary material for: Hypothalamic FTO upregulates BDNF to promote GnRH expression through the PI3K/Akt pathway, leading to precocious puberty
Source: Front Endocrinol (Lausanne). 2025 Oct 31;16:1665391. doi: 10.3389/fendo.2025.1665391 (PMC12615161; doi:10.3389/fendo.2025.1665391)

Uncropped Western Blot Images Corresponding to Figures in the Main Text and Supplementary Materials.

Figure 3B

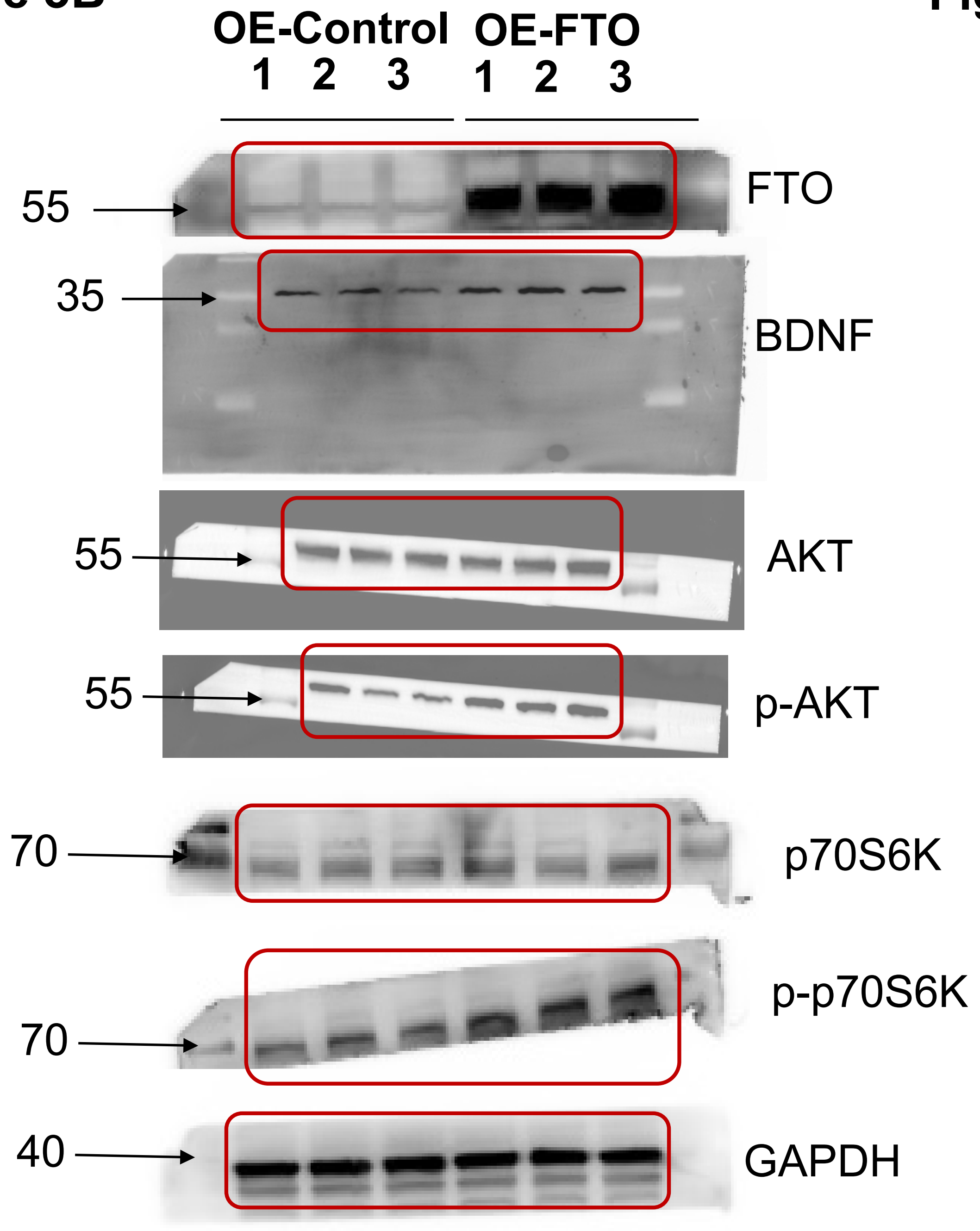

Figure 3D

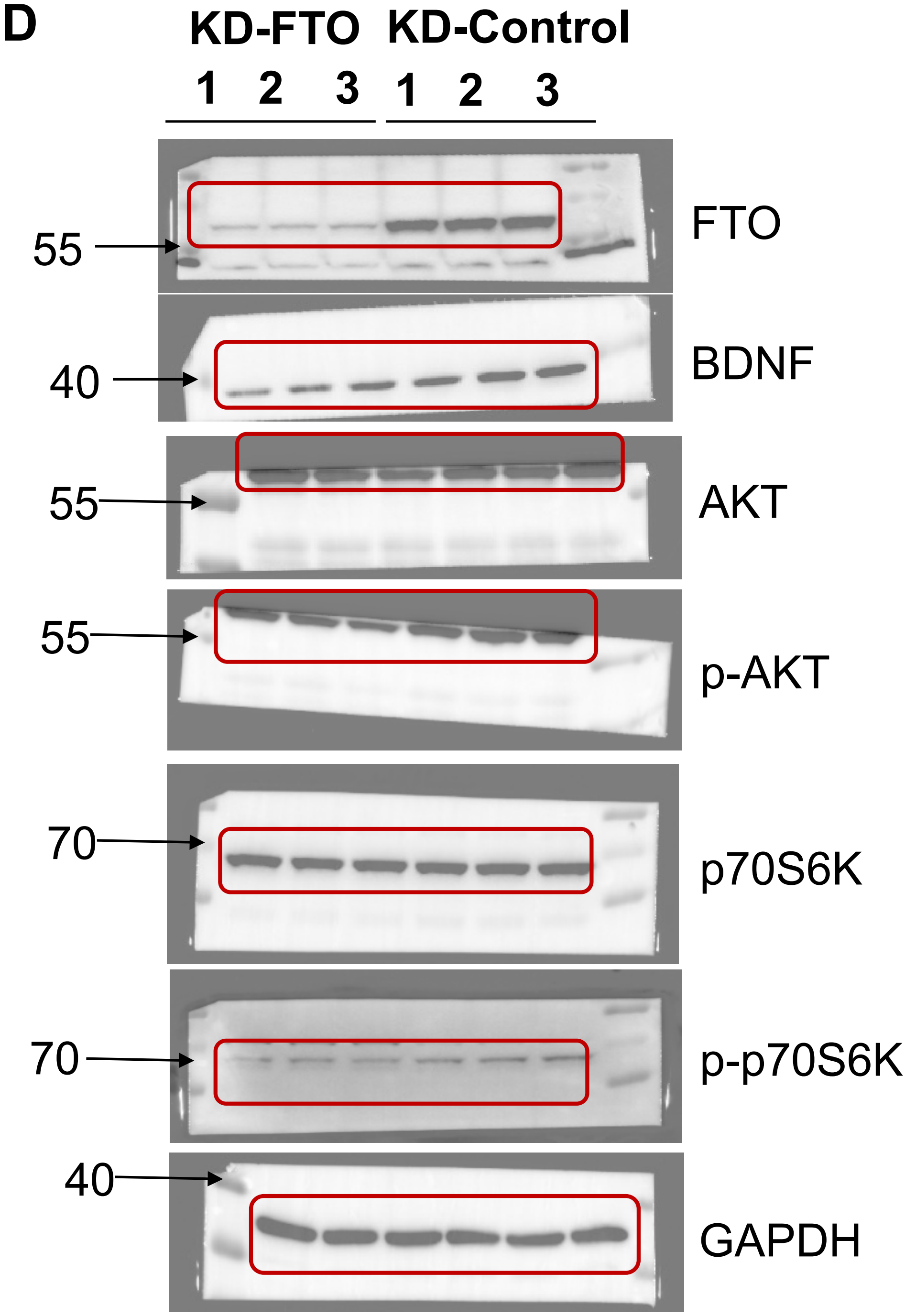

Figure 4B

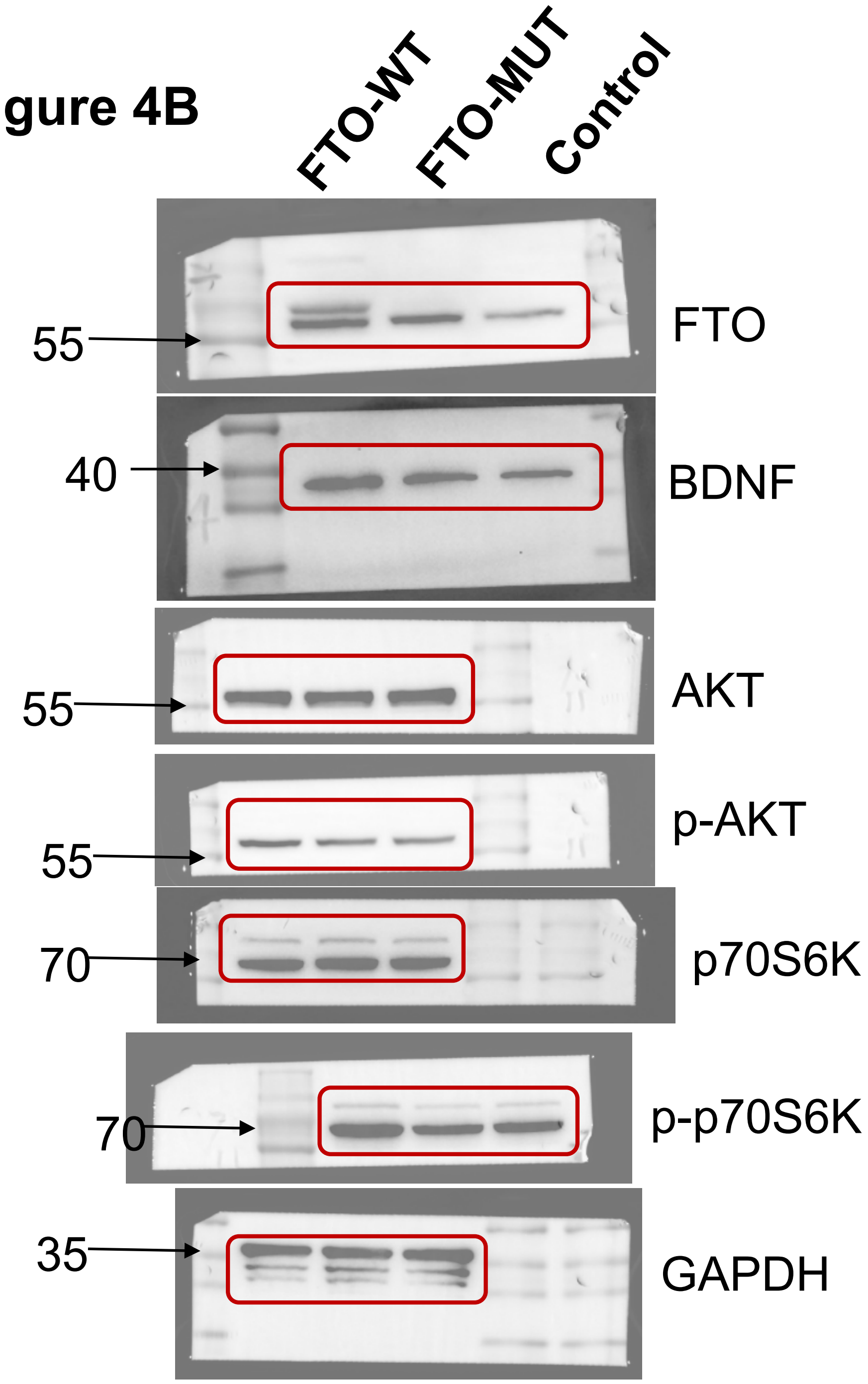

Figure 4E

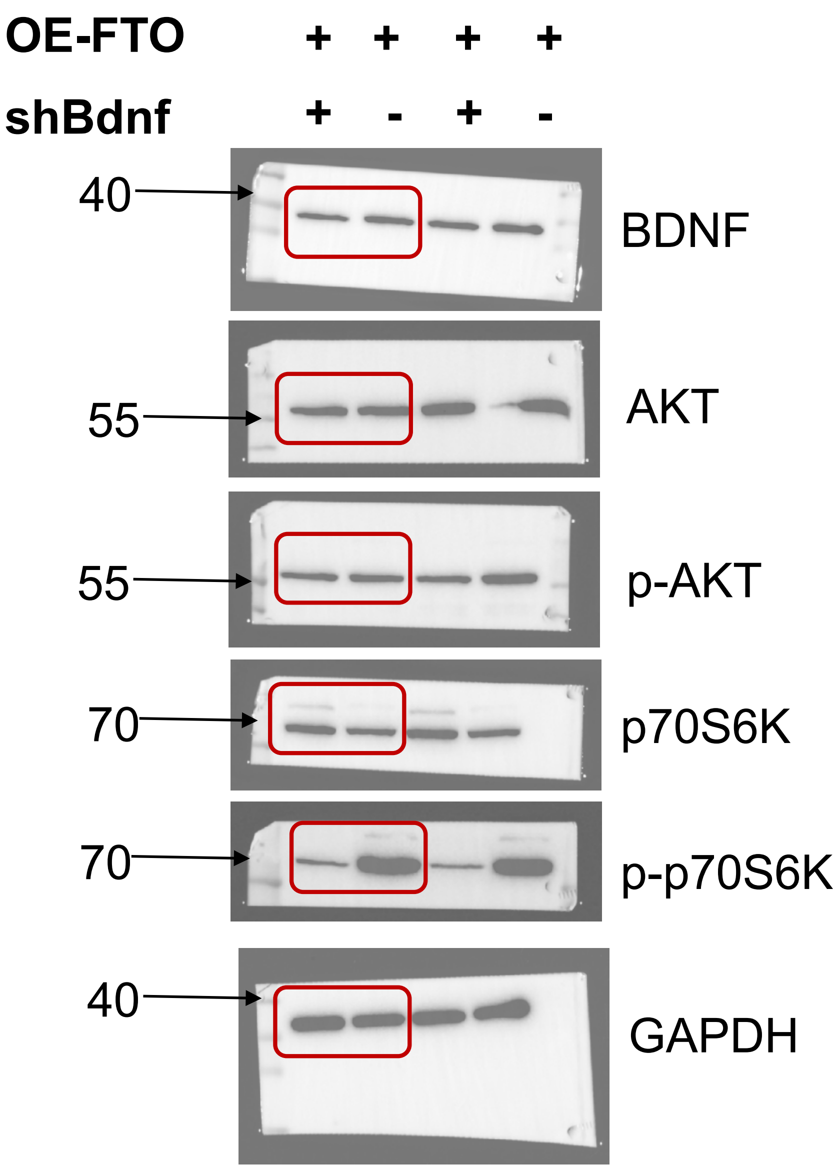

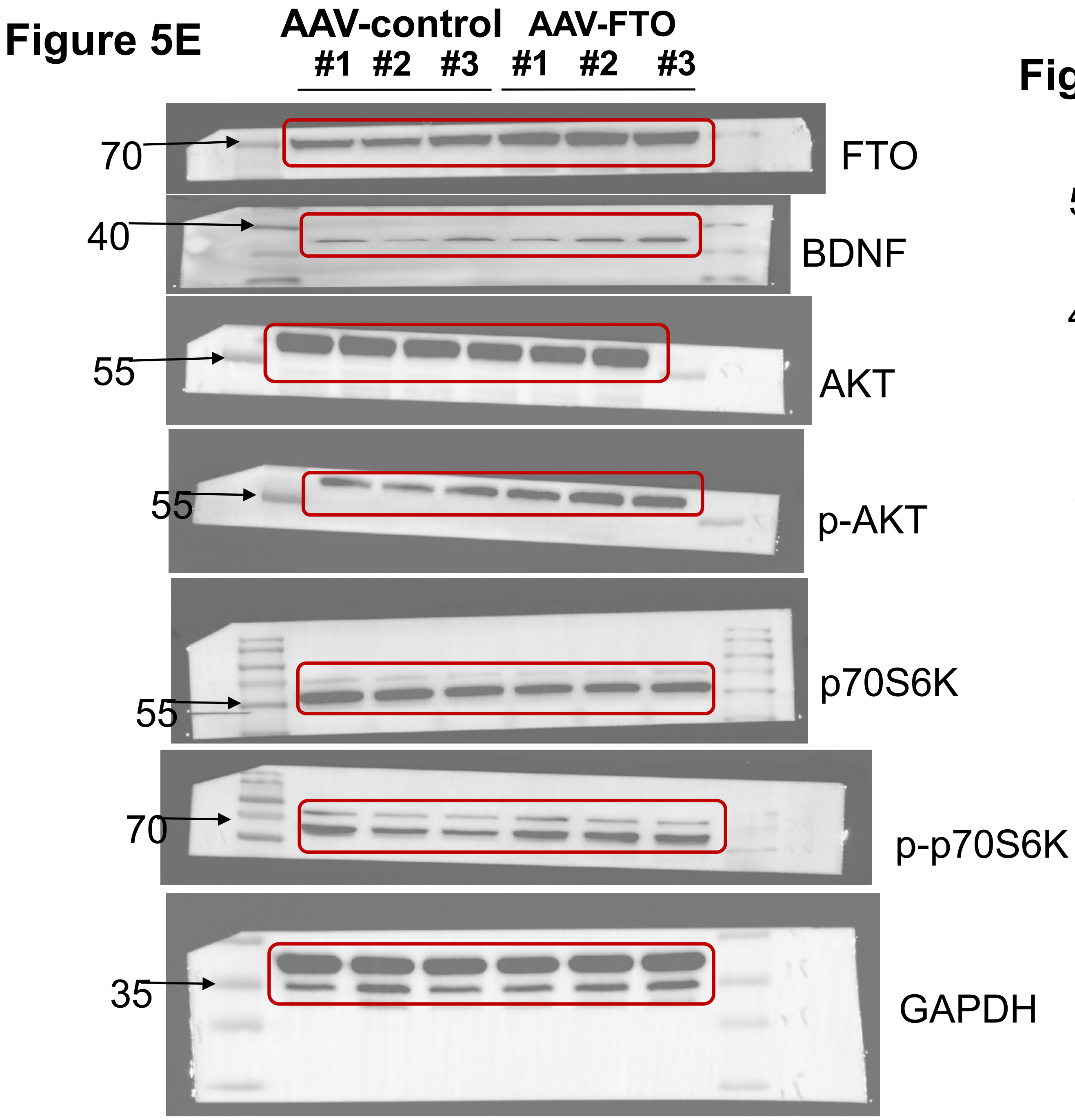
Figure 5E
AAV-control
AAV-FTO
#1 #2 #3 #1 #2 #3
70
FTO
40
BDNF
55
AKT
55
p-AKT
55
p70S6K
70
p-p70S6K
35
GAPDH

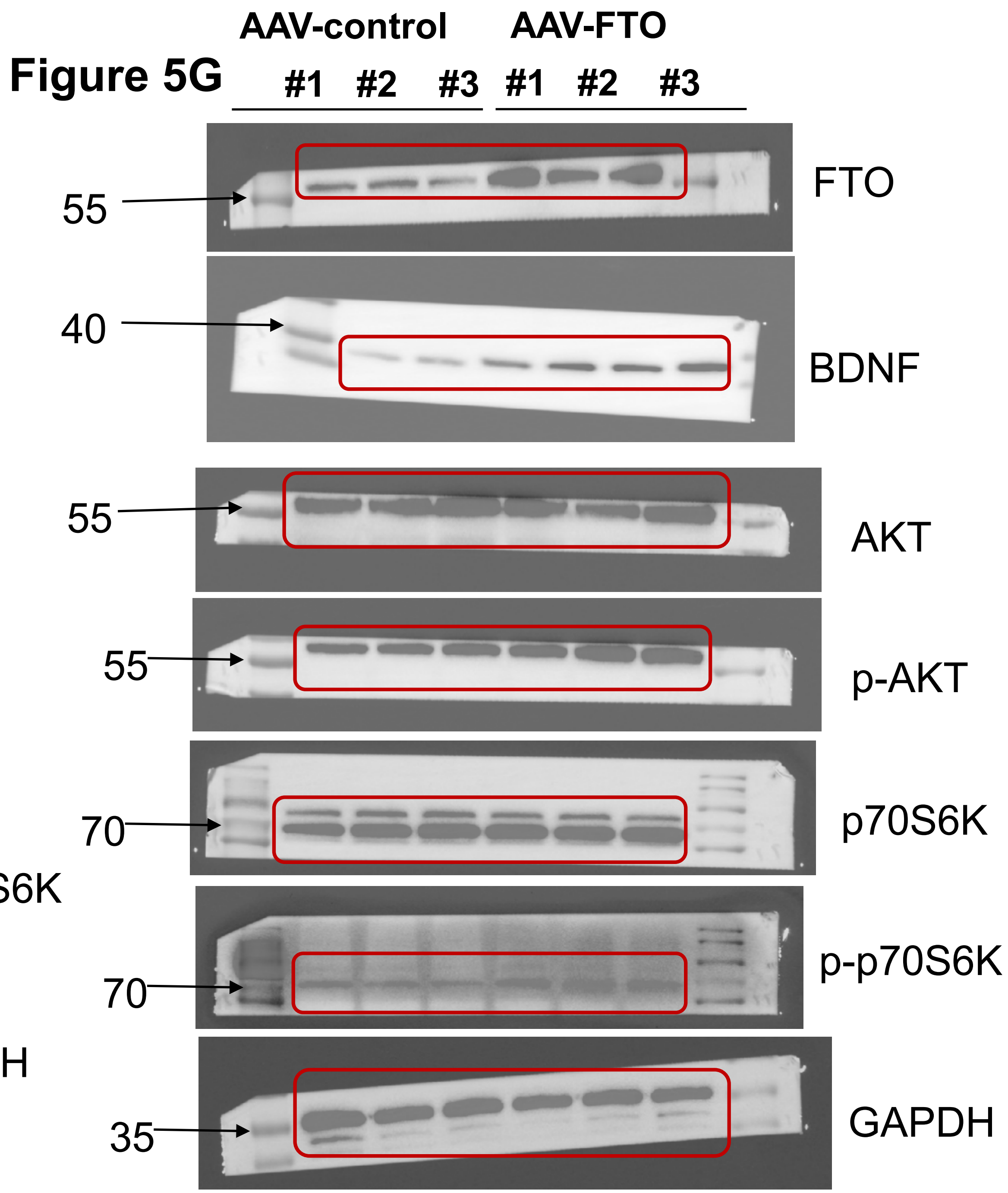
Figure 5G
AAV-control
AAV-FTO
#1 #2 #3 #1 #2 #3
55
FTO
40
BDNF
55
AKT
55
p-AKT
70
p70S6K
70
p-p70S6K
35
GAPDH

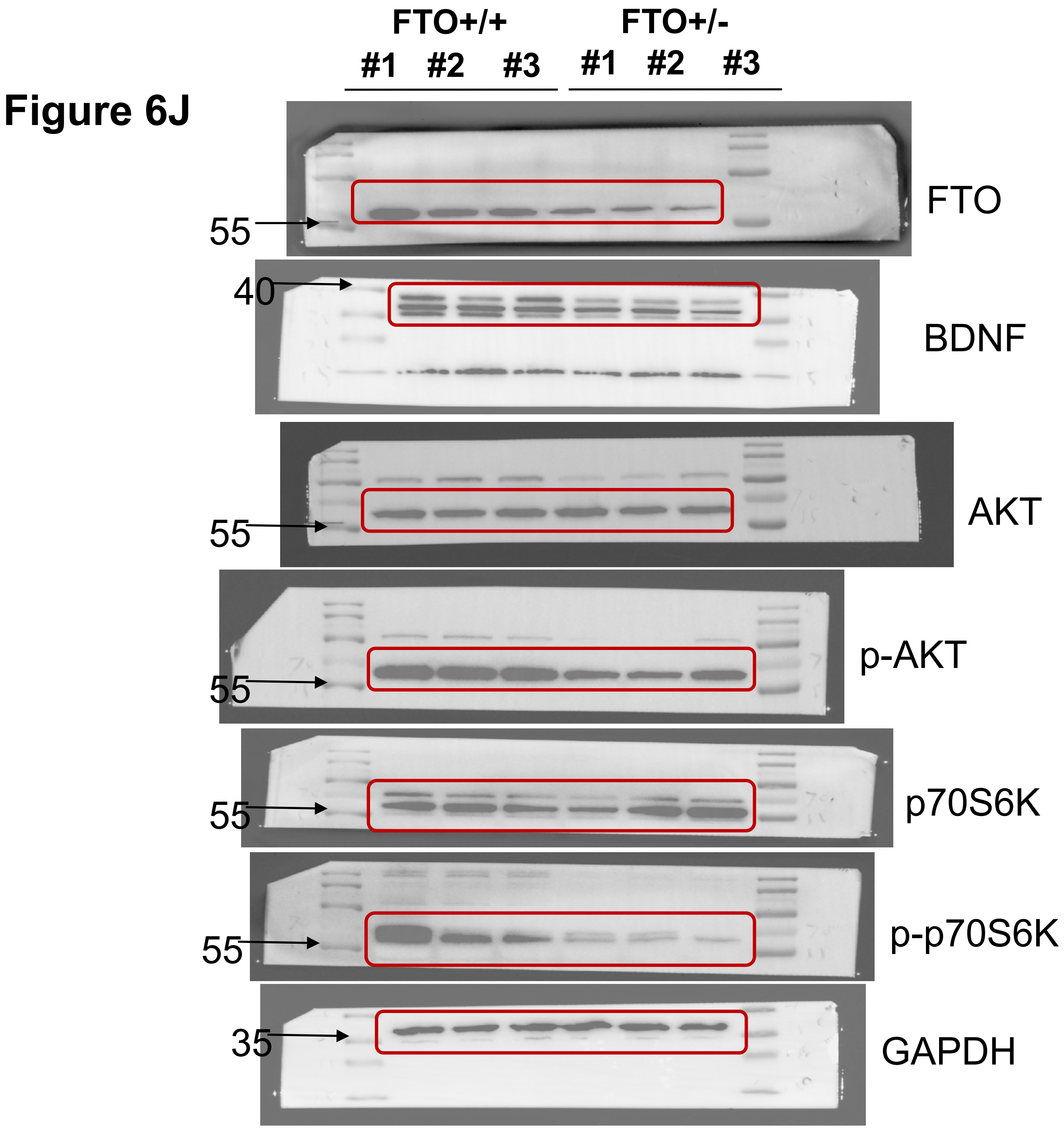
FTO+/+
FTO+/-
#1 #2 #3
#1 #2 #3
Figure 6J
55
FTO
40
BDNF
55
AKT
55
p-AKT
55
p70S6K
55
p-p70S6K
35
GAPDH

Supplementary Figure 1C

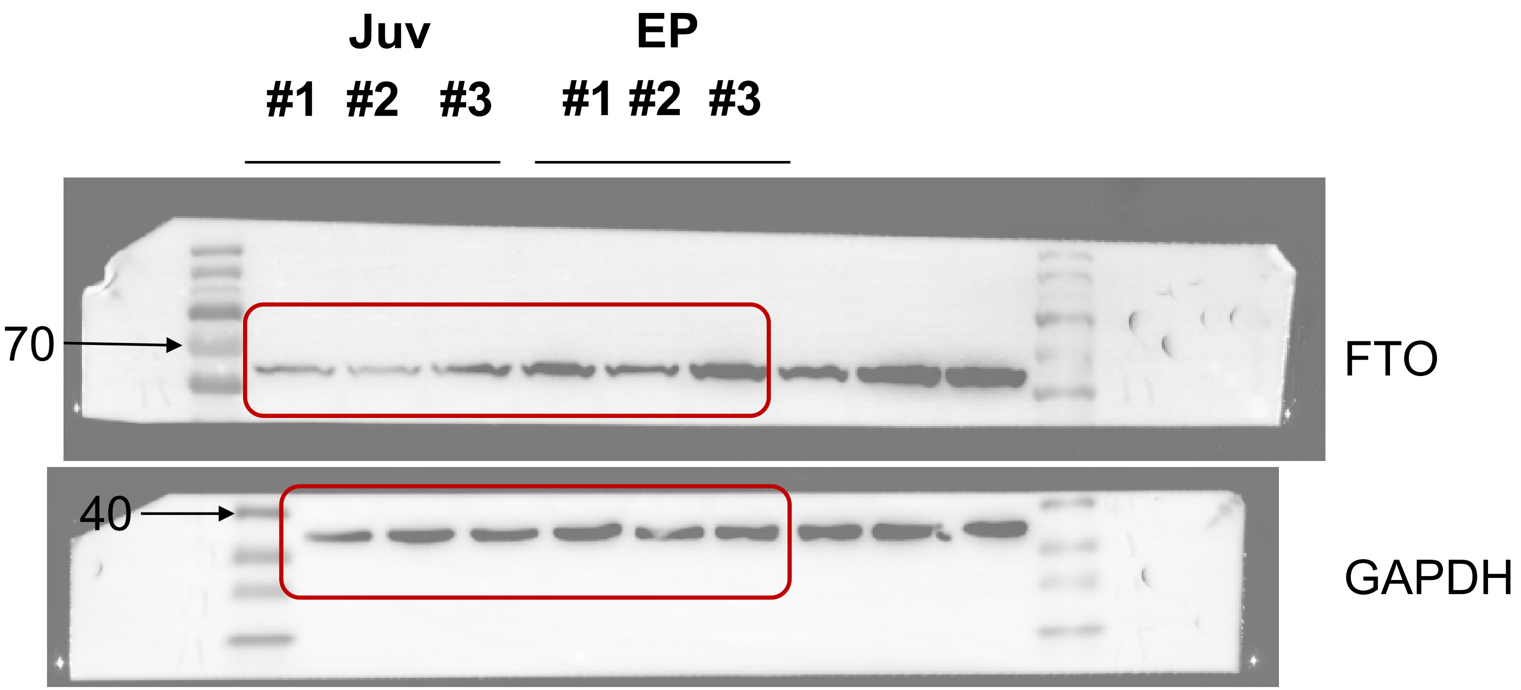

Supplementary Figure 2B

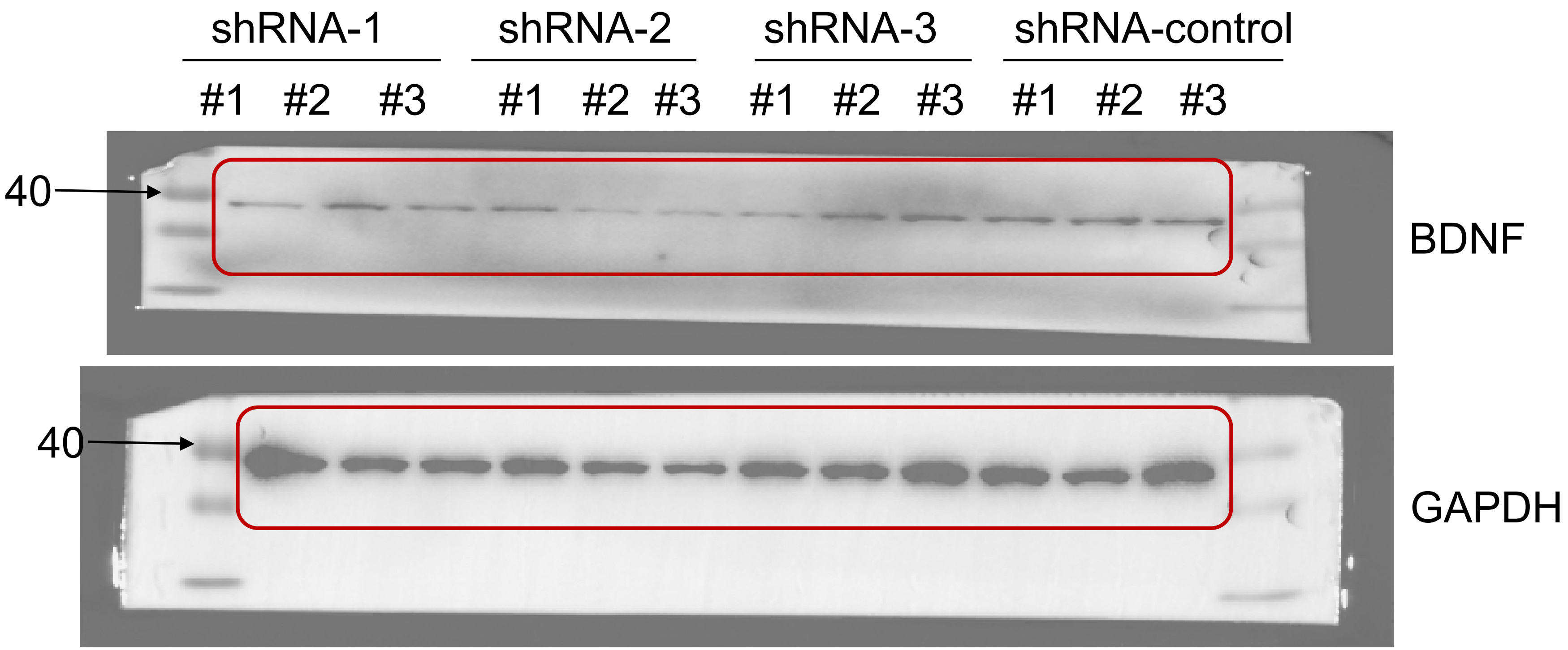

Supplementary Figure 3B

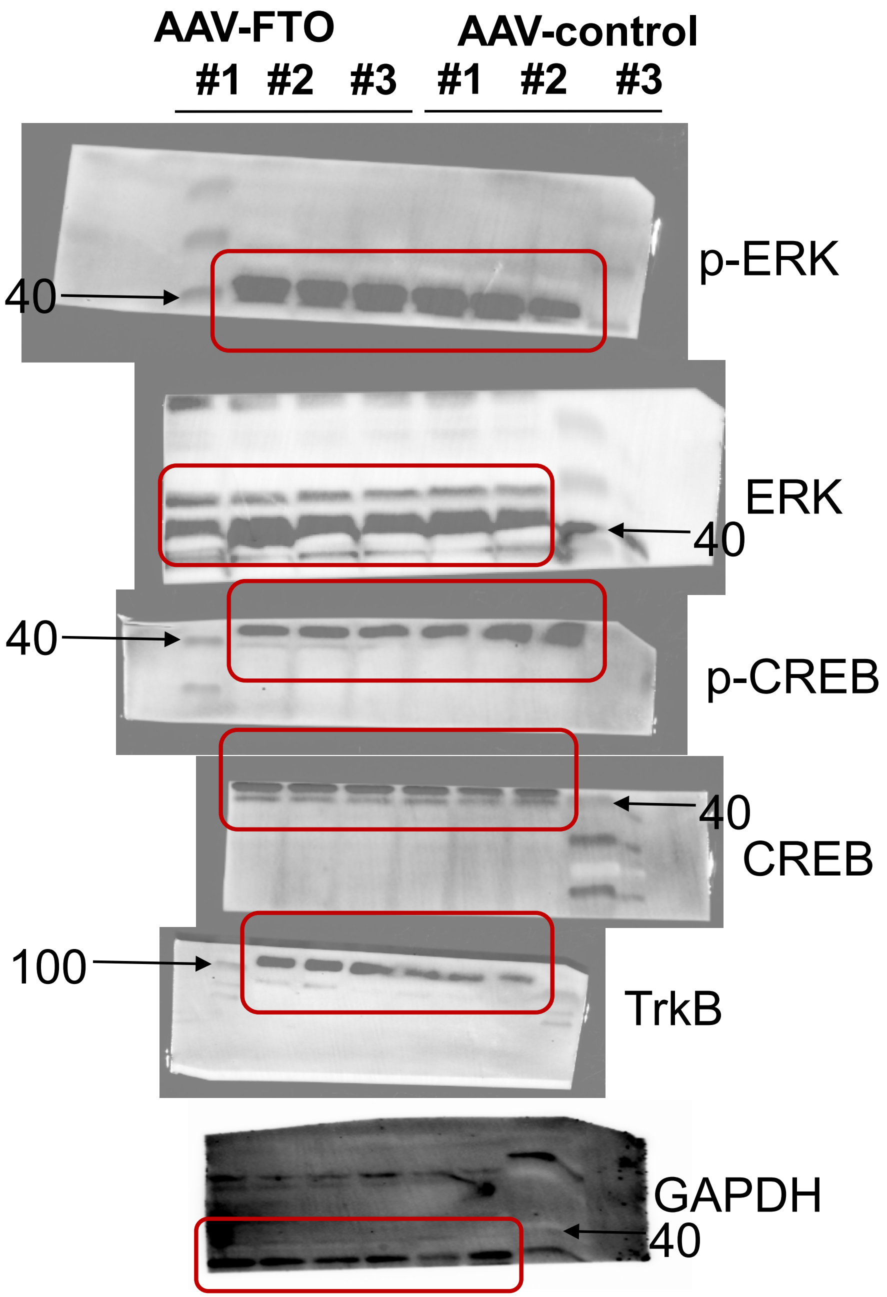

Supplementary Figure 3D

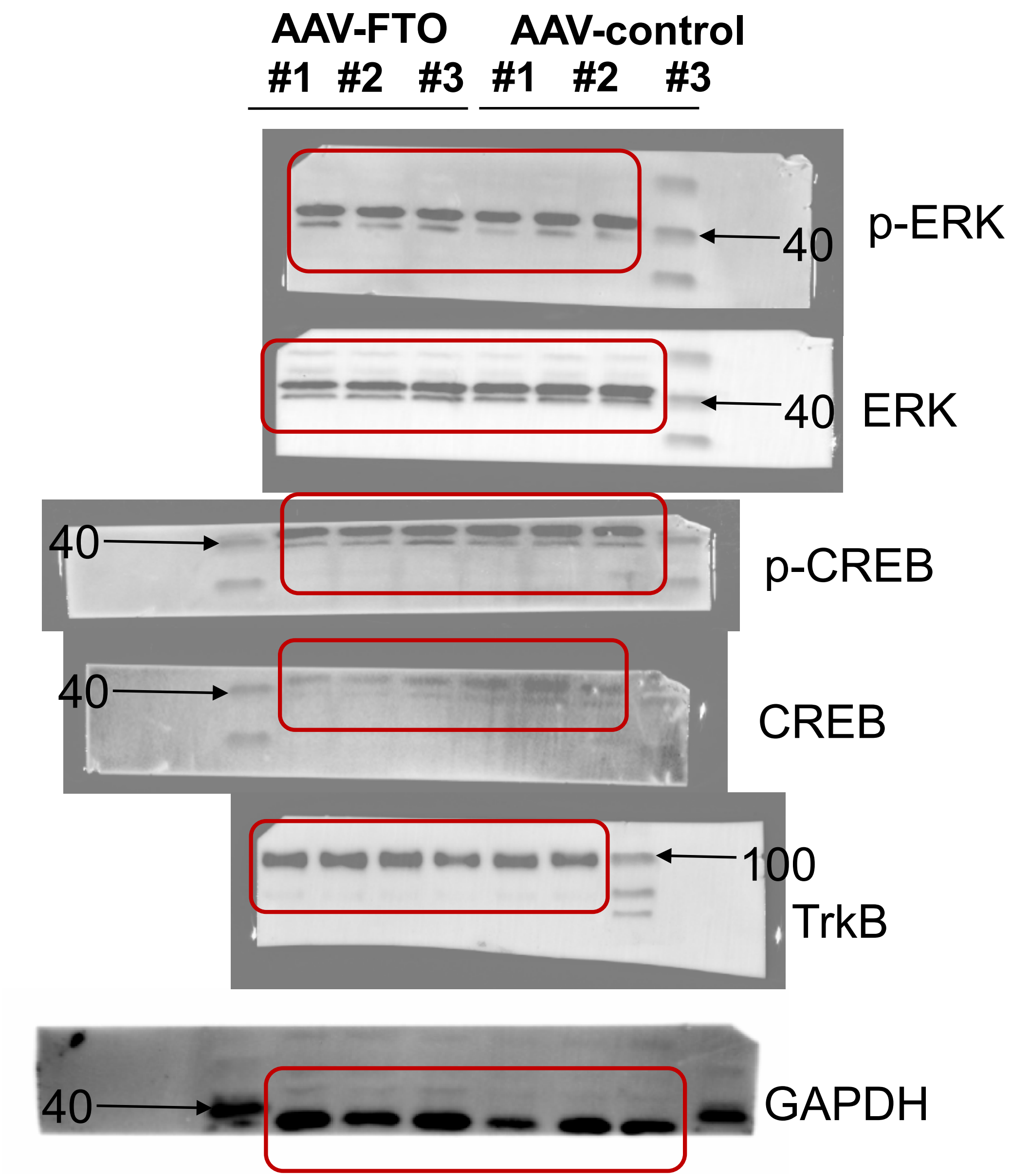

Supplementary Figure 4G

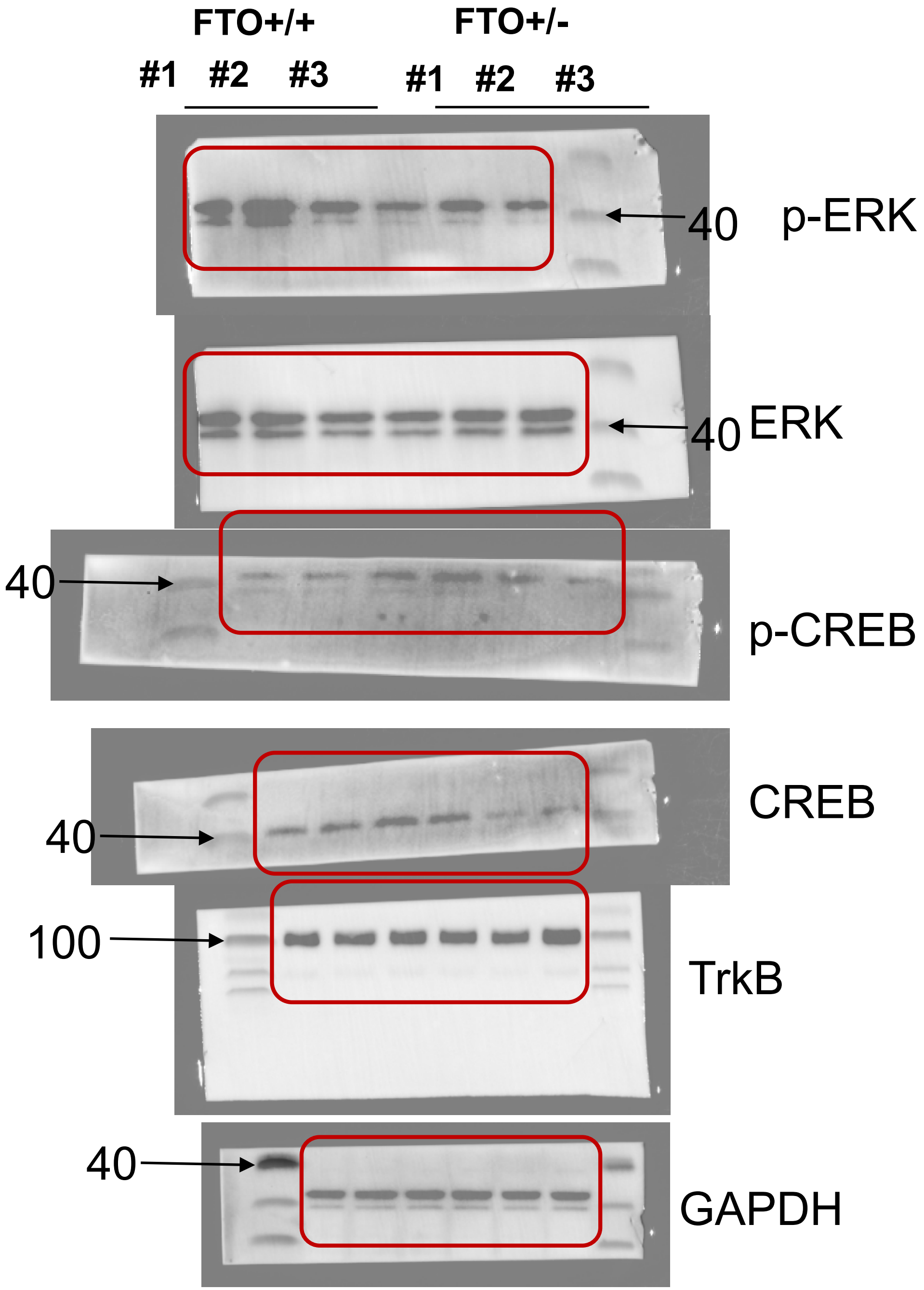

Supplement: Supplementary file 5 [file DataSheet1.pdf]
