# Supplementary material for: Hypothalamic FTO upregulates BDNF to promote GnRH expression through the PI3K/Akt pathway, leading to precocious puberty
Source: Front Endocrinol (Lausanne). 2025 Oct 31;16:1665391. doi: 10.3389/fendo.2025.1665391 (PMC12615161; doi:10.3389/fendo.2025.1665391)
Supplement: Supplementary file 6 [file Table1.docx]

**Supplementary materials**

**Table S1. Sequences of qPCR primers.**

| **Gene** | **Species** | **Forward primer (5’-3’)** | **Reverse primer (5’-3’)** | |
| --- | --- | --- | --- | --- |
| **qPCR primers** |  |  |  | |
| *β-actin* | mouse | AAGATCAAGATCATTGCTCCTCC | GACTCATCGTACTCCTGCTTGC | |
| *FTO* | mouse | GAGCAGCCTACAACGTGACT | GAAGCTGGACTCGTCCTCAC | |
| *GnRH* | mouse | TGATCCTCAAACTGATGGCCG | CGCAACCCATAGGACCAGTG | |
| *Bdnf* | mouse | TCATACTTCGGTTGCATGAAGG | ACACCTGGGTAGGCCAAGTT | |
| *Akt-1* | mouse | ATGAACGACGTAGCCATTGTG | TTGTAGCCAATAAAGGTGCCAT | |
| *Akt-2* | mouse | ACGTGGTGAATACATCAAGACC | GGGCCTCTCCTTATACCCAAT | |
| *Akt-3* | mouse | AGGTTGGGTTCAGAAGAGGG | AGGGGATAAGGTAAGTCCACATC | |
| *Kiss1* | mouse | CTCTGTGTCGCCACCTATGG | AGGCTTGCTCTCTGCATACC | |
| *NKB* | mouse | CTCCTCGTGATGCCCTCTAAT | AGGGAGCAAATCAGGGGGT | |
| *Dyn* | mouse | TGTGCAGTGAGGATTCAGGATG | ACCGTCAGGGTGAGAAAAGATG | |
| *β-actin* | rat | TGCCGCATCCTCTTCCTC | GGTCTTTACGGATGTCAACG | |
| *GnRH* | rat | CCGCTGTTGTTCTGTTGACTGTG | GGGGTTCTGCCATTTGATCCTC | |
| *FTO* | rat | GACACTTGGCTTCCTTACCTG | CTCACCACGTCCCGAAACAA | |
| *Bdnf* | rat | TGTGGTCAGTGGCTGGCTCTC | ACAGGACGGAAACAGAACGAACAG |  |
| *Kiss1* | rat | AGCTGCTGCTTCTCCTCTGT | AGGCTTGCTCTCTGCATACC |  |
| *NKB* | rat | TGATCTCTCTCTGCTACCTCCAC | CCCTGTCTTTATGATGCAGTCC |  |
| *Dyn* | rat | CTCTCCAGCAGGTTTGGC | CTGGGACCGAGTCACCAC |  |
| **MeRIP-qPCR primers** | | |  |  |
| *Bdnf* peak | mouse | AGCAAAGCCGAACTTCTCAC | TTTGCTGTCCTGGAGACTCA |  |

**Table S2. The information of antibodies and reagents used in this study.**

| **Antibodies** | **Company** | **Catalog Number** | **Application** |
| --- | --- | --- | --- |
| GAPDH | CST | #2118 | WB |
| GnRH | Santa Cruz | sc-32292 | IF |
| BDNF | Proteintech | 28205-1-AP | IF |
| FTO | Santa Cruz | sc-271713 | IF |
| FTO | ABclonal | A1438 | WB |
| BDNF | ABclonal | A18129 | WB |
| AKT (pan) | CST | #4691 | WB |
| phosphor-AKT | CST | #4060 | WB |
| p70S6K | CST | #9202 | WB |
| phosphor-p70S6K | CST | #9209 | WB |
| TrkB | Abclonal | A21227 | WB |
| CREB | CST | #9197 | WB |
| Phospho-CREB | CST | #9198 | WB |
| ERK | Abclonal | A4782 | WB |
| Phospho-ERK | Abclonal | AP0974 | WB |
| HRP Goat Anti-Rabbit IgG (H+L) | ABclonal | AS014 | WB |
| HRP Goat Anti-Mouse IgG (H+L) | Abclonal | AS003 | WB |
| Cy3-conjugated Goat anti-Mouse IgG (H+L) | Abclonal | AS008 | IF |
| CoraLite594-conjugated  donkey anti-Rabbit IgG  (H+L) | Proteintech | SA00013-8 | IF |
| FITC-conjugated donkey  anti-mouse IgG (H+L) | Proteintech | SA00003-9 | IF |

**Table S3. Oligonucleotides sequences for short hairpin RNA (shRNA), overexpression and knockdown plasmid, and virus.**

| **Oligonucleotides name** | **Species** | **Sequence (5’-3’)** |
| --- | --- | --- |
| LV-FTO-OE | mouse | NM_011936.2 |
| LV-FTO-KD | mouse | GCTGAGGCAGTTCTGGTTT |
| shRNA-Bdnf-1 | mouse | GAATTGGCTGGCGATTCATAA |
| shRNA-Bdnf-2 | mouse | TTCTACGAGACCAAGTGTAAT |
| shRNA-Bdnf-3 | mouse | TGAGCGTGTGTGACAGTATTA |
| shRNA-control | mouse | CCTAAGGTTAAGTCGCCCTCG |
| scAAV-FTO-OE | rat | NM_001039713.1 |

LV, lentivirus; scAAV, self-complementary adeno-associated virus; OE, overexpression; KD, knockdown.

**Table S4. Clinical characteristics of girl participants with central precocious puberty and control.**

|  | CPP group  (n=14) | Control group  (n=10) | *P* value |
| --- | --- | --- | --- |
| Age (years) | 7.931±0.3335 | 8.02±0.1812 | 0.8356 |
| Height (cm) | 133.7 (129.5-138.3) | 128.5 (124.7-134.0) | 0.0732 |
| Body weight (kg) | 31.47±1.303 | 26.71±2.015 | 0.0314* |
| BMI (kg/m2) | 17.61±0.6143 | 15.60±0.6520 | 0.0382* |
| Tanner stage of breast development | 1.7 (1-2) | 3 (2-4) | <0.001*** |

CPP, Central precocious puberty; BMI, Body mass index.

*Statistically significant.
